# Hidden origami in *Trypanosoma cruzi* nuclei highlights its non-random 3D genomic organization

**DOI:** 10.1128/mbio.03861-24

**Published:** 2025-04-17

**Authors:** Natália Karla Bellini, Pedro Leonardo Carvalho de Lima, David da Silva Pires, Julia Pinheiro Chagas da Cunha

**Affiliations:** 1 Cell Cycle Laboratory, Butantan Institute 196591, São Paulo, Brazil; 2 Center of Toxins, Immune Response and Cell Signaling (CeTICS), Butantan Institute, São Paulo, Brazil; Albert Einstein College of Medicine, Bronx, New York, USA

**Keywords:** *Trypanosoma cruzi*, Hi-C, nuclear architecture, CF unities, TAD boundaries, epigenetic, nonprotein-coding RNA loci

## Abstract

The protozoan *Trypanosoma cruzi* is the causative agent of Chagas disease and is known for its polycistronic transcription, with about 50% of its genome consisting of repetitive sequences, including coding (primarily multigenic families) and non-coding regions (such as ribosomal DNA, spliced leader [SL], and retroelements, etc). Here, we evaluated the genomic features associated with higher-order chromatin organization in *T. cruzi* (Brazil A4 strain) by extensive computational processing of high-throughput chromosome conformation capture (Hi-C). Through the mHi-C pipeline, designed to handle multimapping reads, we demonstrated that applying canonical Hi-C processing, which overlooks repetitive DNA sequences, results in a loss of DNA–DNA contacts, misidentifying them as chromatin-folding (CF) boundaries. Our analysis revealed that loci encoding multigenic families of virulence factors are enriched in chromatin loops and form shorter and tighter CF domains than the loci encoding core genes. We uncovered a non-random three-dimensional (3D) genomic organization in which nonprotein-coding RNA loci (transfer RNAs [tRNAs], small nuclear RNAs, and small nucleolar RNAs) and transcription termination sites are preferentially located at the boundaries of the CF domains. Our data indicate 3D clustering of tRNA loci, likely optimizing transcription by RNA polymerase III, and a complex interaction between spliced leader RNA and 18S rRNA loci, suggesting a link between RNA polymerase I and II machineries. Finally, we highlighted a group of genes encoding virulence factors that interact with SL-RNA loci, suggesting a potential regulatory role. Our findings provide insights into 3D genome organization in *T. cruzi*, contributing to the understanding of supranucleosomal-level chromatin organization and suggesting possible links between 3D architecture and gene expression.

**IMPORTANCE:**

Despite the knowledge about the linear genome sequence and the identification of numerous virulence factors in the protozoan parasite *Trypanosoma cruzi*, there has been a limited understanding of how these genomic features are spatially organized within the nucleus and how this organization impacts gene regulation and pathogenicity. By providing a detailed analysis of the three-dimensional (3D) chromatin architecture in *T. cruzi*, our study contributed to narrowing this gap. We deciphered part of the origami structure hidden in the *T. cruzi* nucleus, showing the unidimensional genomic features are non-randomly 3D organized in the nuclear organelle. We uncovered the role of nonprotein-coding RNA loci (e.g., transfer RNAs, spliced leader RNA, and 18S RNA) in shaping genomic architecture, offering insights into an additional epigenetic layer that may influence gene expression.

## INTRODUCTION

*Trypanosoma cruzi* is a heteroxenic protozoan parasite and the etiological agent of Chagas disease, which primarily affects Latin American countries. However, it has now reached a global distribution, impacting nonendemic regions as well (1). Genomic peculiarities of *T. cruzi* include directional clusters of genes transcribed polycistronically by RNA polymerase II without well-known promoter regions (except for spliced leader [SL] genes) (2); the absence of introns (except for the poly-A polymerase gene) (3); RNA processing mediated by the trans-splicing machinery (4); an abundance of repetitive DNA within coding (multigenic families, MF) and non-coding (mainly retroelements, satellite DNA, ribosomal, and SL) sequences (5–7); and the linear genome compartmentalization into core regions, which contain conserved syntenic genes, and disruptive regions, which harbor genes encoding surface-associated virulence factors from MFs and nonsyntenic genes (trans-sialidases, mucins, and mucin-associated proteins [MASPs]), with MFs as Gp63, dispersed gene family 1 (DGF-1), and retrotransposon hot spot (RHS) (hereafter collectively referred to as the GpDR group) distributed across both regions (7).

Expression of proteins in *T. cruzi* can be controlled at various levels. Most eukaryotes mainly regulate gene expression at transcription initiation, while trypanosomatids rely predominantly on post-transcriptional mechanisms, including transcript processing, stability, translation (4), and codon usage bias (8, 9). Nonprotein-coding RNAs (ncRNAs) are involved in post-transcriptional regulation of gene expression, contributing to post-transcriptional control mechanisms. As in other eukaryotes, small nuclear RNAs (snRNAs) are required for mRNA maturation and consist of U-rich snRNAs that form the spliceosome and SL-RNAs that provide the 39 nt sequence for trans-splicing (10). Ribosomal RNAs (rRNAs) are extensively processed and chemically modified during their maturation, which are important for their function in the ribosome (11). In addition, both SL-RNA loci and rRNA loci have been associated with three-dimensional (3D) nuclear compartmentalization in trypanosomatids and in mammals, respectively, impacting gene expression (12–14).

Epigenetic factors such as nucleosome occupancy and dynamics (15), open chromatin status (16), DNA modifications such as base J deposition at the transcription termination sites (TTS) (17, 18), histone variant deposition (19, 20), and histone post-translational modifications (21) have also been demonstrated to be additional players in gene regulation in trypanosomes. In *T. cruzi*, epigenetic mechanisms have been investigated in replicative and nonreplicative forms. The infective, nonproliferative form shows lower global transcriptional levels of RNA polymerase II associated with nuclear chromatin alterations (22). The chromatin structure is dynamically modulated in its different life forms, with open chromatin in epimastigotes enriched in core genes and closed chromatin associated with genes encoding virulence factor proteins (disruptive genes) (16). Histone variants such as H2B.V are found at transcription start sites (TSSs) and often colocalize with transfer RNA (tRNA) loci (19), while H4.V is enriched at TTS and telomeric regions (Rosón J. N. et al. 2024, unpublished data). During differentiation, trypomastigotes exhibit increased heterochromatin and a redistribution of euchromatin, primarily concentrated in the inner nucleus. This is accompanied by increased nucleosome density at TSS regions, which correlates with a global reduction in transcription levels (15, 16).

3D nuclear architectures, such as active and inactive compartments, are linked to gene expression control (23, 24) and to DNA replication and repair (25). This 3D chromatin organization within cell nuclei, similar to origami, adopts diverse and intricate structures. Linear DNA, primarily packed in chromatin fibers, folds into distinct 3D chromatin architectures, with the two most well-characterized being topologically associated domains (TADs) and chromatin loops (26) (see Fig. 8). As in origami, folding exposes certain regions while hiding others, enabling distant DNA loci to come into proximity for interactions, such as enhancers looping to promoters (27, 28). These 3D arrangements, shaped by protein‒DNA interactions and chromatin remodelers, are essential for gene regulation, genome stability, and maintaining functional chromatin architecture (29, 30).

Chromosome conformation capture (3C), introduced by Dekker et al. in 2002 (31), enabled the study of 3D DNA‒DNA interactions within the nucleus. This technique involves cross-linking DNA, digesting it with an endonuclease, and ligating it to preserve 3D contacts, followed by PCR to detect interactions. The method, adapted from nuclear ligation assays by Cullen et al. in 1993 (32), was further advanced by Lieberman-Aiden et al. in 2009 to become the Hi-C method, which uses next-generation sequencing to map genome-wide DNA interactions (23). In trypanosomatids, the Hi-C technique was first utilized by Muller et al. to reveal the 3D nuclear organization of core and subtelomeric regions and centromeres (33). A subsequent study revealed spatial proximity between active variant surface glycoprotein (VSG) expression sites and SL-RNA loci, suggesting that switching among VSG expression sites is linked with their proximity to the SL array in *Trypanosoma brucei* (12). In 2021, Hi-C was employed for the first time in a *T. cruzi* strain (Brazil clone A4) to aid genome assembly by resolving gaps in short-read and long-read sequences (34), and further used to highlight the organization of core and disruptive genomic compartments (35).

Chromatin interactions are potentially encoded in the DNA sequence through a sophisticated interplay of protein binding sites and various sequence elements. Many algorithms have been developed to predict genome folding based on its own sequence (36, 37). DNA motifs associated with certain architectural proteins such as CCCTC-binding factor (CTCF) proteins, promoters, enhancers, repetitive sequences, and nonprotein-coding RNA genes have been shown to play critical roles in 3D genome architecture (38–40). Despite the valuable contributions in understanding the 3D chromatin structure of core and disruptive compartments in *T. cruzi* (35), the study did not address the genomic features nor the roles of non-coding protein genes in the 3D nuclear architecture, or the impact of discarding multimapped reads—often associated with repetitive regions in this analysis. As a result, a significant portion of the *T. cruzi* genome was excluded from previous 3D nuclear architecture analyses. The repetitive nature of the *T. cruzi* genome (6, 41) poses substantial challenges for conventional Hi-C processing pipelines (42–44), which typically exclude multimapped reads due to their association with low-quality mappings and multiple alignments. These reads predominantly originate from repetitive genomic regions, including surface-associated virulence factors from MF members presented at the disruptive/GpDR compartment, ribosomal RNA loci, and SL regions, among others, in *T. cruzi*. Here, we aimed to address the abovementioned gaps by applying a robust pipeline (45) that incorporates high-quality multimapped reads into 3D nuclear architecture analyses, using a publicly available Hi-C data set from the Brazil A4 strain (29). By including these reads, we improved the DNA–DNA contact maps and uncovered previously undescribed 3D interactions involving non-coding RNA loci, thereby broadening our understanding of the *T. cruzi* 3D genome structure.

## RESULTS

### Comprehensive *T. cruzi* genomic annotation and multimapping strategies

To assess the 3D genome structure of *T. cruzi,* including repetitive regions, we first improved genome annotation and repeat analysis. Loci for small nucleolar RNAs (snoRNAs), snRNAs, tRNAs, SL-RNAs, and rRNAs were unannotated in the available genome of the *T. cruzi* Brazil A4 strain (34). Using an in-house pipeline, we identified 1,199 snoRNA genes, 105 ncRNA genes (SLs and snRNAs which comprise the uridine-rich small nuclear RNA genes), and 201 rRNA (18S, 5S, 5.8S, and 24S) genes (Table S1). For these analyses, although we used non-stringent BLASTn search parameters, our approach was based on pre-existing annotations in *T. cruzi*. Therefore, we cannot rule out the possibility that other non-coding regions could be identified using alternative approaches. Additionally, we identified a total of 71 tRNA genes whose results proved to be as sensitive as the tRNAScan output, detecting the same number of tRNA genes at identical genomic positions. Moreover, our approach exhibits sensibility precision, as we were able to identify four undetermined tRNA genes by tRNAScan, revealing them as valine tRNAs (Table S2). By identifying the orientation of both Box A and Box B for each tRNA gene, we generated a map of its distribution in the *T. cruzi* Brazil A4 genome, in which it is evident that tRNA genes tend to form clusters composed of two or more genes in arrays (Fig. S1). Additionally, we identified 1,364 polycistronic units (PTUs), accompanied by 407 convergent strand switch regions (cSSRs), 416 divergent strand switch regions (dSSRs), and 445 intergenic regions (IRs).

**Fig 1:**
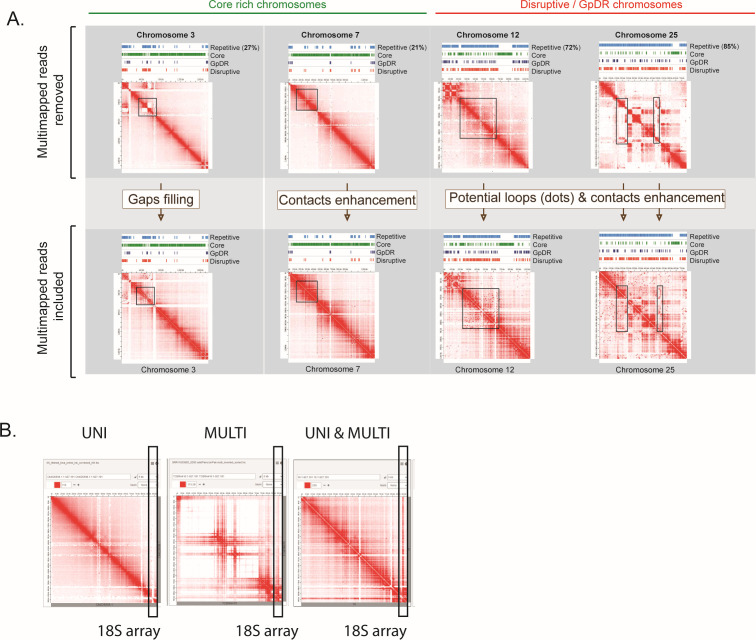
Inclusion of multimapped reads in Hi-C data analysis provides an enhancement of Hi-C contacts. (A) Juicebox view of individual Hi-C matrices obtained excluding multimapped reads via the HiCExplorer pipeline (upper panel). The inclusion of multimapped reads from the mHi-C pipeline is shown in the lower panel. The black rectangles highlight DNA‒DNA contacts and loop enhancement, and gap filling recovered to the repetitive regions (light blue tracks). DNA loops are commonly seen in Hi-C matrices as punctual dots, potentially enriched for disruptive regions of the genome (Chr 12 and Chr 25). Tracks seen above the individual matrices indicate multigenic families located in the *GpDR* (dark blue), disruptive (red) compartment, and core (green) compartment. (B) Hi-C contacts enhancement for the 18S array using the mHiC tool (UNI & MULTI), third matrix. UNI (first) and MULTI (second) matrices were generated using exclusively single-mapped and multimapped reads, respectively.

We computed the proportion, in terms of length, per chromosome of the linear genome organization (core/disruptive/*GpDR* genes), repetitive DNA, and pseudogenes (Fig. S2). Core genes are more prevalent in the *T. cruzi* Brazil A4 genome, which comprises 76.2% (11,515 out of 15,111 total genes), while disruptive and *GpDR* genes constitute 16.63% (2,512 genes) and 7.17% (1,084 genes) of the genome, respectively (Fig. S2B).

Repetitive DNA is prevalent over the 43 chromosomes, particularly in those with higher concentration of *GpDR* and disruptive genes (Fig. S2A through C). The top 5 repetitive-rich chromosomes are Chr 35, 36, 38, 39, and 42 (Fig. S2C). Chromosomes with high repetitive DNA content usually have increased number of pseudogenes (Fig. S2C). Nonprotein-coding genes account for approximately 0.2% of all repetitive DNA (Fig. S3A). Within this class, the SL and 5S gene sets exhibit a high degree of conservation, whereas the conservation among tDNA and snoDNA genes is very low (with yellow nucleotides indicating >90% conservation, as shown in Fig. S3B). Additionally, no similarity was detected within cSSR and dSSR regions. These comprehensive genomic annotations and characterizations played a pivotal role in our analysis of the 3D genome structure of *T. cruzi*, as shown below.

Traditional Hi-C analysis pipelines typically exclude reads that map to multiple genomic locations, relying solely on uniquely mapped reads, as in the analyses conducted by reference 35. In this study, we sought to incorporate high-quality multimapped reads into our 3D nuclear architecture analyses. To that, we employed the multimapping strategy for Hi-C data analysis (mHiC tool) which optimizes the allocation of multimapped reads (45), previously used in *T. brucei* (12). This pipeline assigns Hi-C reads to a single location in the genome in a non-random manner, selecting the most likely site based on the average interaction profile derived from the intrinsic properties of Hi-C read pairs (45). In parallel, we compared the results obtained using the pipeline that excludes multimapped reads (HiCExplorer tool) (44), as used previously in 3D analysis in *T. cruzi* (35).

The inclusion of high-quality multimapped reads allowed us to allocate more than 12 million additional reads compared to traditional Hi-C pipelines (Table S3). Discarding multimapped reads in traditional pipelines introduces gaps in Hi-C matrices (Fig. 1A, upper panel), which can obscure critical DNA‒DNA contacts, potentially leading to erroneous biological conclusions. This issue is particularly pronounced in chromosomes enriched in disruptive genes, such as chromosomes 12 and 25, which consist of 72% and 86% repetitive DNA sequences, respectively (Fig. 1A). Figure 1A illustrates the comparison between these two strategies (HiCExplorer × mHiC), highlighting the improvements in gap filling, contact enhancement, and potential loop detection in four *T. cruzi* chromosomes by mHi-C. By accounting for multimapped reads, we can now assess the 3D interactions occurring at the 18S rRNA loci (Fig. 1B) and SL loci as shown below. This finding underscores the importance of including multimapped reads to reveal previously unknown DNA‒DNA contacts within the *T. cruzi* genome.

### Chromatin-folding domains and chromatin loops reflect the linear genomic organization of core and disruptive compartments

To evaluate the 3D genome structure of *T. cruzi*, we used a TAD caller algorithm to identify TAD-like domains (here named as chromatin-folding [CF] domains) using Hi-C matrices of various resolutions. We found that lower-resolution matrices resulted in fewer TAD-like regions with longer median lengths (Fig. S4A and C), suggesting a hierarchical organization with nested CF domains (Fig. S4D). The median CF domain length of 43 Kbp in 5 Kbp resolution Hi-C matrices (Fig. S4C) is significantly shorter than the 880 Kbp median TAD length observed in mammals (46). Higher resolution matrices (2 Kbp–5 Kbp) lead to CF domains that cover a greater percentage of the genome (68%–79%) (Fig. S4B), comparable to the 90% coverage in mammals (46). Therefore, we focused on matrices with 2 Kbp–5 Kbp resolution for further analysis.

We characterized CF domains based on the unidimensional genomic composition of *T. cruzi* (core and disruptive compartments, in addition to *GpRD* genes). We examined whether these linear compartments also formed distinct 3D compartments. Our analysis revealed that 51% of the CF domains were mixed (containing genes from more than one compartment), while 49% were pure (containing genes from only one compartment) (data not shown). Pure CF domains composed of core genes were longer (4 to 178 Kbp) than those composed of disruptive and *GpDR* genes (4 to 28 Kbp) (Fig. S5). This variation in CF length suggests that CF domains enriched in disruptive/*GpDR* genes, which are shorter than the core CF domains, are also more compact in the nucleus, as recently suggested (35).

**Fig 2:**
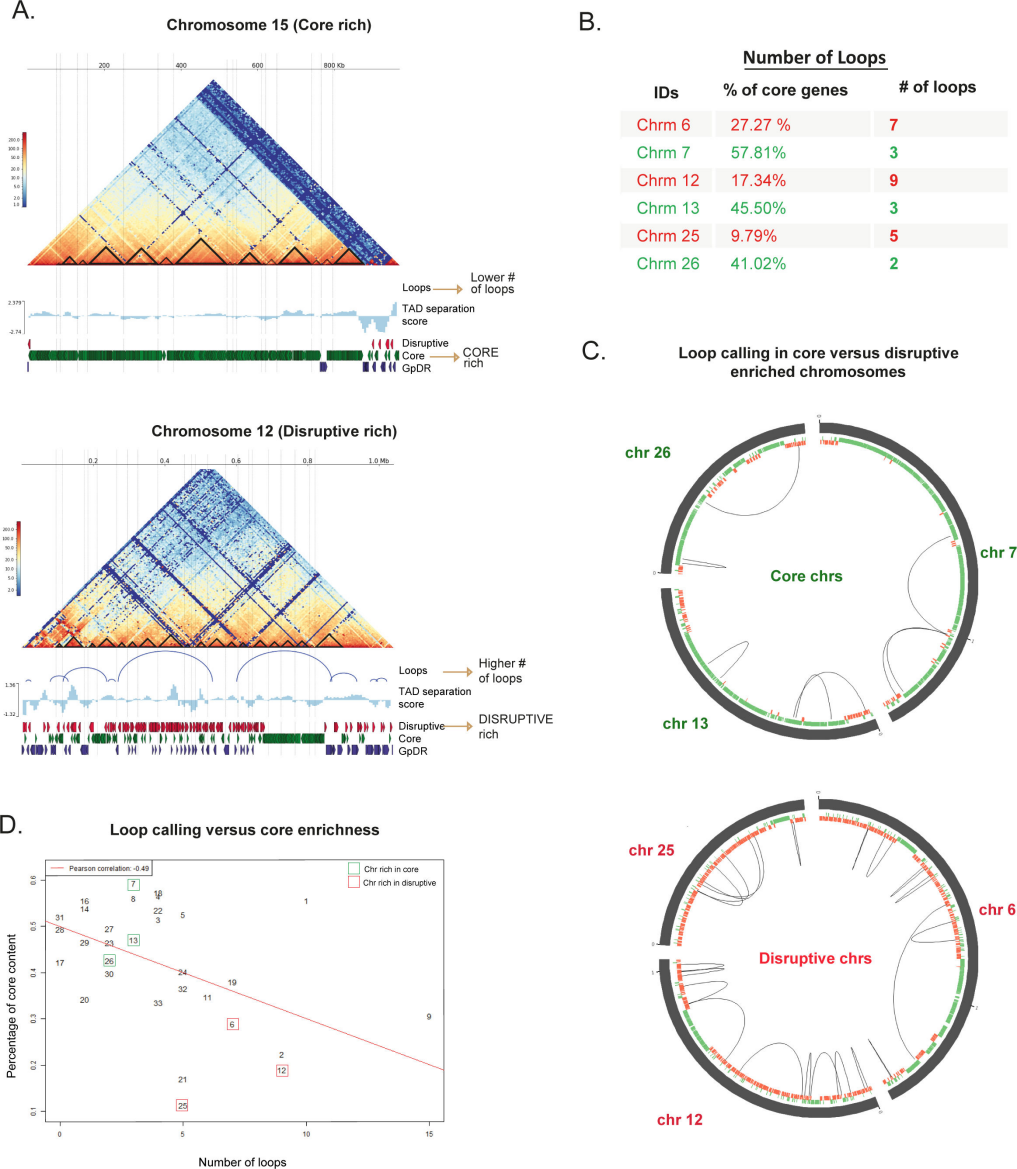
The core-rich chromosomes contain fewer DNA loops than disruptive/*GpDR*-rich ones. (A) Knight-Ruiz-normalized Hi-C heatmaps with the identification of loops (blue semicircles—x-axis) for a core-rich chromosome (chr 15) and a disruptive-rich chromosome (chr 12). (B) Analysis of chromosome pairs with differing core gene contents (e.g., chromosomes 6 and 7, 12 and 13, and 25 and 26). Chromosomes enriched in core genes (chr 7, 13, and 26) displayed fewer loops than did those enriched in disruptive genes (chr 6, 12, and 25). (C) Loop calling for core and disruptive enriched chromosomes. The Circos plot (https://circos.ca/) was used to draw the interactions. (D) Dot plot showing the number of loops (*x*-axis) per numbered chromosome. There is a negative correlation (*R* = −0.5) between the percentage of core genes (*y*-axis) and the number of loops (*x*-axis) across all 43 chromosomes, indicating that loops in *T. cruzi* are influenced by the linear genomic organization into core and disruptive compartments.

To investigate the impact of these linear compartments on intrachromosomal interactions, we compared the number of chromatin loops in chromosomes enriched in the core, disruptive, and *GpDR* genes. Notably, chromatin loops were more frequent in chromosomes enriched in disruptive/*GpDR* genes than in those enriched in core genes (Fig. 2). For instance, chromosome 15, which is enriched in core genes (~62.3%), had no loops, whereas chromosome 12, which is impoverished in core genes (~17.3%), contained nine loops (Fig. 2A). This pattern was also observed for chromosome pairs 6 and 7, 12 and 13, and 25 and 26, which have similar lengths but differ in core gene enrichment (Fig. S2D). Chromosomes enriched in core genes (chr 7, 13 and 26) had at least half the number of loops compared to those enriched in disruptive genes (chr 6, 12, and 25) (Fig. 2B and C). We observed that the majority (15/29; 51.7%) of interactions occur between genes within the same compartment while 7% (2/29) occur between compartments. Interestingly, 41.3% of loops (12/29) were found between a defined compartment (either core or disruptive) and genomic regions located between compartments.

Overall, we observed a negative correlation (*R* = −0.5) between the percentage of core genes and the number of loops across all 43 chromosomes (Fig. 2D). In summary, the CF domains and chromatin loops in *T. cruzi* reflect the linear genomic organization into core and disruptive compartments, influencing the 3D genome structure and interactions.

### Impact of multimapped reads detection on CF domain identification

**Fig 3:**
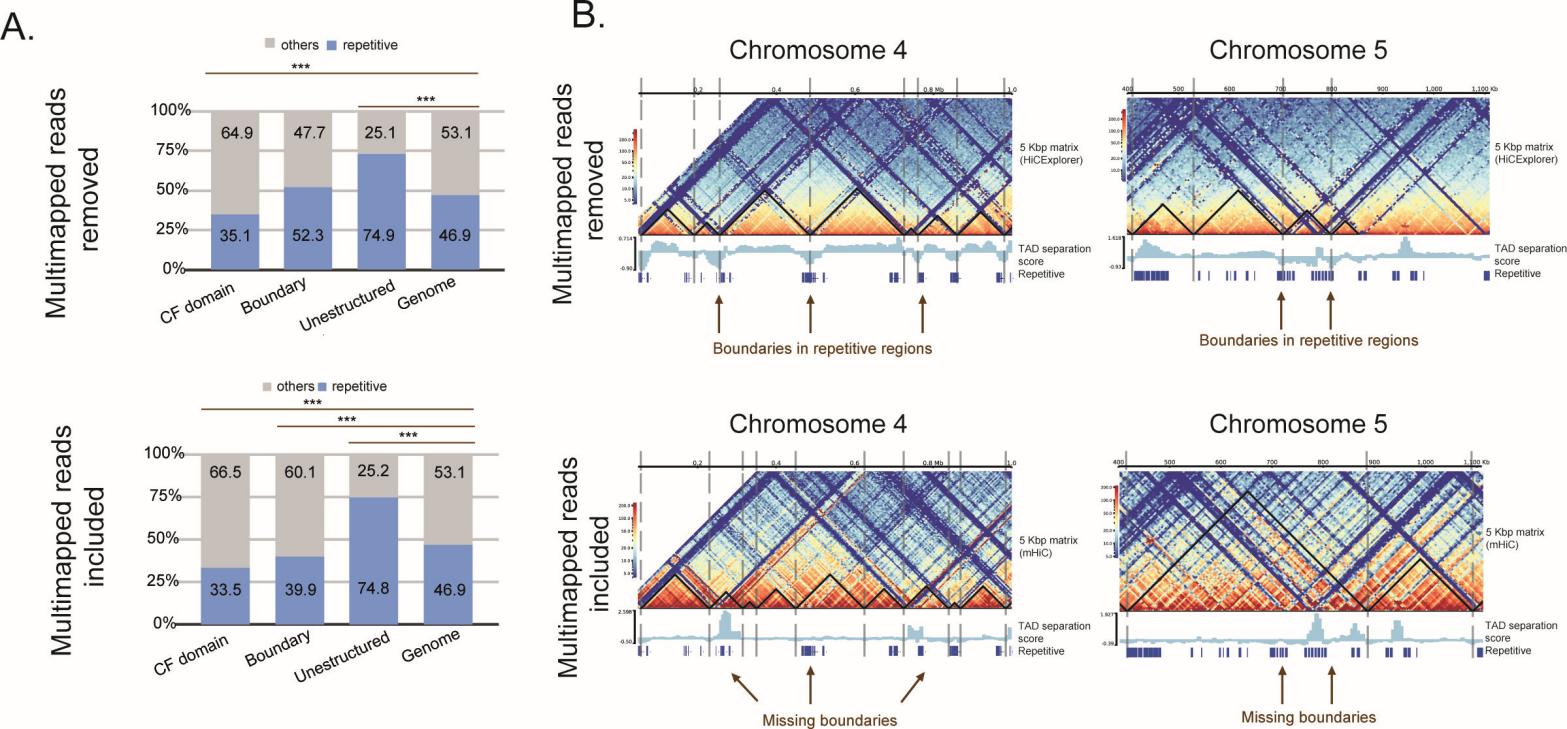
Repetitive DNA in Hi-C data analysis mitigates bias in CF calling and its boundaries. (A) Distribution of repetitive DNA (light blue) and nonrepetitive DNA (gray) within CF domains, boundaries, unstructured regions, and the genome (control) compared with the results obtained when processing Hi-C data excluding multimapped reads, HiCExplorer, upper panel, or including it, mHiC, lower panel, pipelines. *** *P*-value < 0.01 using the χ^2^ test (False Discovery Rate [FDR]-adjusted *P*-values). (B) Shifts in TAD boundaries (vertical dashed lines) and CF domains (black triangles). The exclusion of repetitive reads causes an enrichment of CF domain boundaries in repetitive regions (e.g., chromosomes 4 and 5), while their inclusion shifted some boundaries to nonrepetitive regions, indicating biases in TAD calling due to the exclusion of multimapped reads.

Next, we evaluated the impact of including or discarding multimapped reads (mainly from repetitive regions) on the identification of 3D genome structures, specifically CF domains, their boundaries, and unstructured regions. To do this, we compared the distribution of repetitive DNA within these structures against their expected distribution in the genome (Fig. 3A). Notably, the classification of regions as unstructured remains unaffected by whether the multimapped reads are included or not. These unstructured regions contain more than 70% repetitive DNA—a topic that will be explored later.

Excluding multimapped reads from the Hi-C data analysis resulted in the identification of 212 CF domains and 250 boundaries (Fig. S6A). The inclusion of multiple mapped reads yielded similar numbers, with 203 CF domains and 242 boundaries (Fig. S6A), as well as comparable median CF domain length (Fig. S6B).

Notably, CF domain boundaries exhibited approximately 52% enrichment in repetitive DNA when multimapped reads were excluded—similar to the genome distribution. Conversely, when multimapped reads were included, the repetitive DNA content at CF boundaries decreased to approximately 40% (Fig. 3A). Further examination of CF boundaries across 20 chromosomes (Chr 1 to 20) confirmed that the inclusion of multimapped reads reduced the number of boundaries within repetitive regions and increased the percentage of boundaries detected in nonrepetitive DNA regions (Fig. S6C). Specifically, excluding multimapped reads highlighted CF boundaries enriched in repetitive DNA on chromosomes 4 and 5. In contrast, including multimapped reads caused a shift, with certain boundaries previously identified in repetitive regions no longer evident (Fig. 3B). This result suggests that TAD calling is biased by the absence of Hi-C contacts in repetitive regions due to the exclusion of multimapped reads (e.g., using the HiCExplorer pipeline, 34% of the mapped reads were discarded; Table S3). In other words, CF boundaries are not preferentially located within repetitive regions in *T. cruzi*. Without multimapped reads, the percentage of repeats within boundaries matches the percentage observed in the genome. However, when multimapped reads are included, boundaries tend to be located in regions impoverished of repetitive DNA. Across all 3D units analyzed here, the distribution of DNA repeats differs significantly from a random genomic distribution, suggesting potential biological relevance. Ultimately, these findings reinforce the importance of considering repetitive regions to accurately determine 3D genomic structure and avoid erroneous conclusions. Consequently, the analyses described below were performed with the inclusion of multimapped reads.

### CF domains, boundaries, and unstructured regions are characterized by distinct genomic features in *T. cruzi*

**Fig 4:**
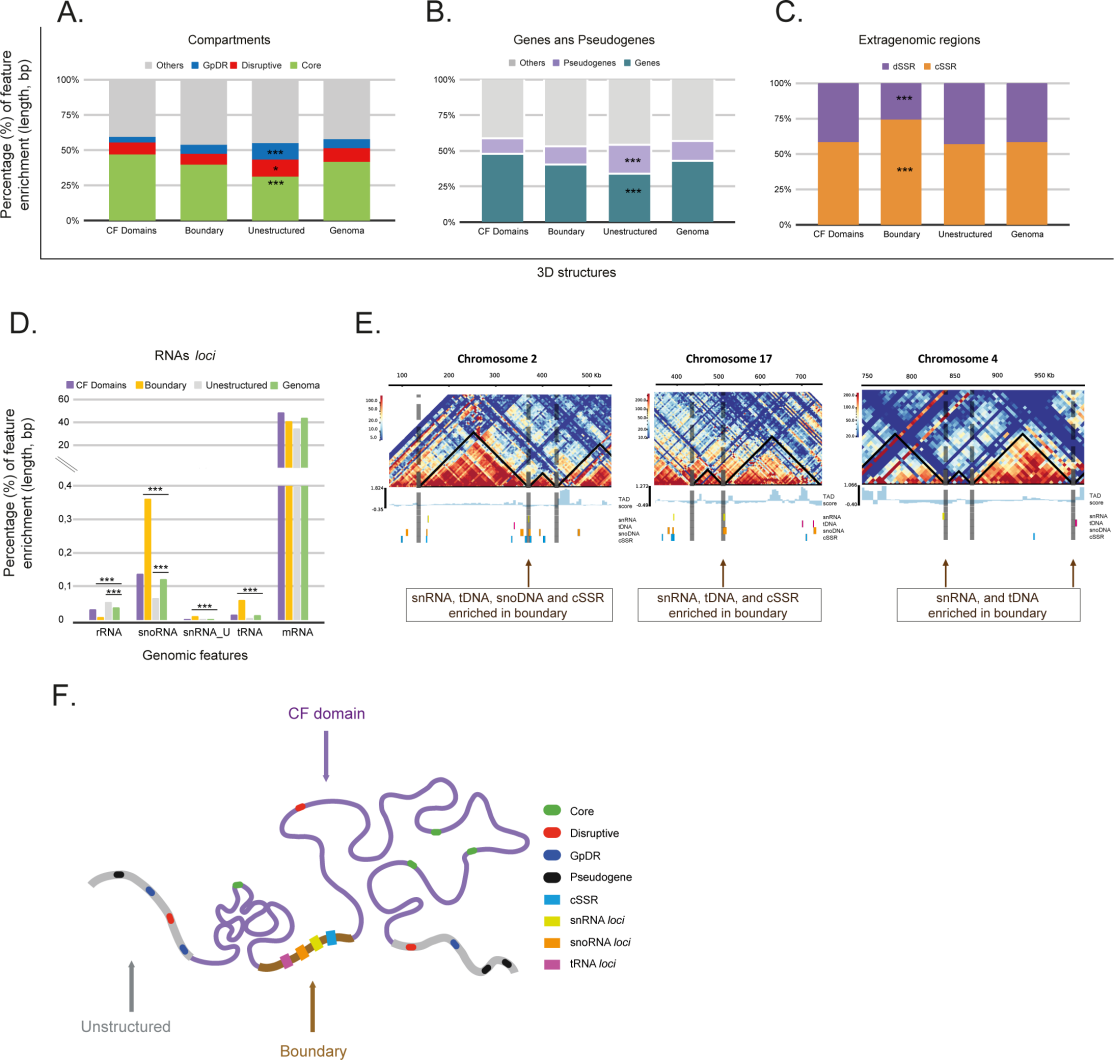
The non-random positioning of *T. cruzi* genomic features along CF domains, their boundaries, and unstructured regions. (A) Distribution profile of core, disruptive, and *GpDR* genes across 3D structures (CF domains, boundaries, and unstructured regions) compared to the genome as a control. *GpDR* (blue) genes and disruptive (red) compartments are enriched in unstructured regions. The others (gray) represent all genomic sequences that remained after excluding the core, disruptive, and *GpDR* sequences. (B) Distribution of genes and pseudogenes across the 3D structures. Pseudogenes (light purple) show a predominance in unstructured regions. The others (gray) represent all the genomic sequences that remained after excluding genes and pseudogenes. (C) Distribution of extragenomic regions, specifically cSSR and dSSR sites, with cSSR sites predominantly located at boundaries. (D) Distribution of small RNA genes (rRNA, snoRNA, snRNA_U, tRNA, and mRNA) across 3D genomic structures. Notably, snoRNA, snRNA_U, and tRNA loci are preferentially located at CF domain boundaries. For A to D, the χ^2^ test indicates significance for False Discovery Rate FDR-adjusted *P*-values <0.05. From A to D, the percentages represent the abundance of each feature (in length, bp) divided by the length of each 3D compartment, or by the entire genome length, for the controls. (E) Co-localization of snRNAs, tRNAs, snoRNA genes, and cSSR at CF domain boundaries for chromosomes 2, 17, and 4. (F) Model illustrating the preferential positioning of target genomic features within the 3D nuclear architecture.

Next, we evaluated whether the 3D genome organization in *T. cruzi*, particularly CF domains and their boundaries, was associated with the enrichment of specific genomic features. By comparing the observed versus expected distributions of certain loci in CF domains, boundaries, and unstructured regions, we noticed that unstructured regions were enriched with genes from the MF (disruptive and *GpDR* genomic compartments) but had fewer core genes (Fig. 4A). MF genes are enriched in unstructured regions, coinciding with chromosome ends, as exemplified in chromosomes 5, 7, and 19 (Fig. S7). These regions are also enriched in pseudogenes (Fig. 4B), aligning with the higher percentages of pseudogenes in the *GpDR* (82.7%) and disruptive compartments (61.5%) (Fig. S2B).

We asked whether each PTU would be folded into a single or multiple CF structures and vice versa. We identified three scenarios: PTUs folded into more than one CF structure; PTUs formed by a unique CF structure; and CF structures covering more than one PTU. Considering that PTUs are delimited by dSSRs and cSSRs, which demarcate the start and stop points of RNA polymerase II transcription, respectively, we evaluated their enrichment in 3D structures. In absolute numbers, 30 cSSRs (8%) and 17 dSSRs (5.5%) coincided with CF boundaries. Thus, the majority of PTU borders (SSRs) do not coincide with CF boundaries. However, considering that CF boundaries cover only 3.5% (in length) of the genome, the presence of SSRs in these regions may be functionally relevant. Considering the relative length of each 3D structure to the entire genome, cSSRs were notably enriched at CF boundaries and were 1.27 times more frequent than they were in the genome-wide distribution (Fig. 4C). This result suggests that some boundaries may impact transcription initiation and termination.

We also investigated whether loci encoding various classes of RNAs (mRNAs, tRNAs, snoRNAs, snRNAs, SL-RNAs, and rRNAs) were associated with specific 3D genome structures. We found significant enrichment of tRNA, snoRNA, and snRNA loci at CF boundaries (Fig. 4D and E). Considering only the group of RNA loci, the tRNA loci made up 7.7% of the genome by length and comprised 13.4% of CF boundaries, indicating a 1.74-fold enrichment. Similarly, the enrichment of the snRNA and snoRNA loci at CF boundaries was 1.17 and 1.85 times greater, respectively, than that at random genomic distribution. These findings were consistent across Hi-C matrices at the 10 Kbp resolution (data not shown), underscoring the significance of these genomic features in the 3D organization of the *T. cruzi* genome. Notably, the loci of cSSRs, snRNAs, snoRNAs, and tRNAs together account for less than 6% of the repetitive sequences (Fig. S3A), suggesting that their preferential location at CF boundaries may have functional significance. A summary of the non-random distribution of each feature is presented in the model shown in Fig. 4F.

### The tRNA loci interactions suggest the formation of a spatial cluster within *T. cruzi* nuclei

**Fig 5:**
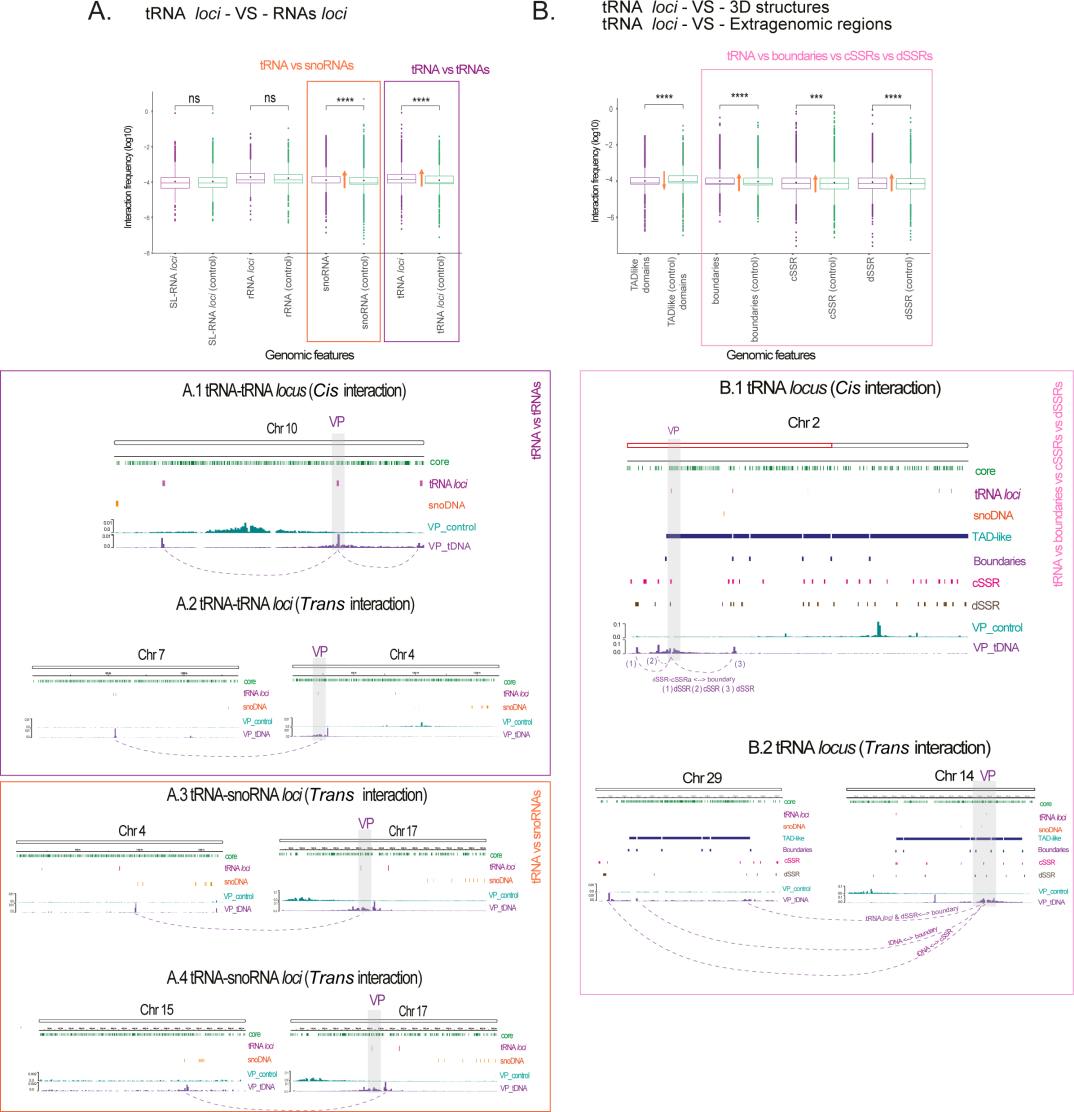
Virtual 4C analysis of tRNA loci as VPs. Panels (**A**) and (**B**) display the comparison of DNA‒DNA interaction frequencies (log10) for tRNA loci with RNA loci and 3D genomic structure, respectively. For each target (purple boxplots), their control counterparts (green boxplots) are compared. Significant differences are indicated by ***P < 0.001 and ****P < 0.0001 (for False Discovery Rate FDR-adjusted *P*-values). Rectangles in orange, purple, and rose indicate genomic features that have a greater frequency of contacts with tRNA genes than with their respective controls. The orange arrows indicate whether the interactions observed for VPs are above or below the control interactions. (A) Integrative Genomics Viewer snapshots depicting *cis*-acting, intra, (A.1) and *trans*-acting, inter, (A.2) chromosomal contacts between the tRNA locus (VP) and other tRNA loci. *Trans*-acting (A.3 and A.4) interactions between the tRNA locus (VP) and snoRNA locus. (B) Significant interactions highlight that DNA‒DNA contacts are frequent between tRNAs and CF domain boundaries in addition to the extragenomic regions cSSRs and dSSRs (rose rectangle). *Cis*-acting (B.1) and *trans*-acting (B.2) chromosomal contacts among the tRNA locus (VP), 3D structures, and extragenomic regions.

Previously, we found that the chromatin of tRNA loci is developmentally regulated (16). Here, we discovered that CF boundaries are enriched in tRNA loci. To provide further insights into the role of tRNA loci in 3D genome organization, we used the virtual 4C approach to screen DNA‒DNA interactions mediated by all tRNA loci (viewpoints, VPs) (Fig. 5). Remarkably, the interactions between tRNA loci (Fig. 5A, tRNA-vs*-*tRNA panel) are stronger than those between randomly selected control loci (tRNAs-vs-controls, boxplots in green). This suggests that some tRNA loci form a 3D cluster through *cis-* and/or *trans*-acting interactions. tRNA genes also exhibit a greater frequency of contact with snoRNAs, which are transcribed by RNA pol II (Fig. 5A, tRNA-vs-snoRNA panel). In contrast, significant interactions were not detected between tRNA loci and SL-RNA loci (also transcribed by RNA polymerase II) or between tRNA loci and rRNA loci (with 24S, 5.8S, and 18S rDNAs transcribed by RNA polymerase I and 5S rDNA genes transcribed by RNA polymerase III). snoRNAs and tRNA genes share a widespread genomic distribution, being the most prevalent nonprotein-coding RNA loci across the *T. cruzi* genome, found on more than 12 different chromosomes (Table S1). Both are enriched in CF boundaries, suggesting that the interaction between tRNAs and snoRNAs may facilitate the grouping of CF boundaries, promoting 3D contacts.

In terms of 3D structures, tRNA loci preferentially interact with CF boundaries and both dSSRs and cSSRs—compared to the control regions used as VPs (Fig. 5B). *Cis*-acting and *trans*-acting interactions represent DNA‒DNA contacts captured between tRNAs, 3D structures (boundaries), and extragenomic regions (cSSRs and dSSRs). Together, these results indicate that tRNA loci perform a vast set of 3D interactions, which include the following: the tRNAs themselves, the snoRNAs, TAD boundaries, cSSRs, and dSSRs. Collectively, these results demonstrate that tRNA loci engage in a broad network of 3D interactions, highlighting their significance in the spatial organization of the *T. cruzi* genome.

### Remarked 3D nuclear networks involve the SL-RNA and 18S rRNA loci in *T. cruzi*

In order to further understand how 3D local interactions take place within the nucleus, we extended our analysis to additional nonprotein-coding RNA loci, emphasizing SL-RNA and rRNA genes. These loci have been previously reported as important contributors to 3D nuclear organization (12, 47–49).

**Fig 6:**
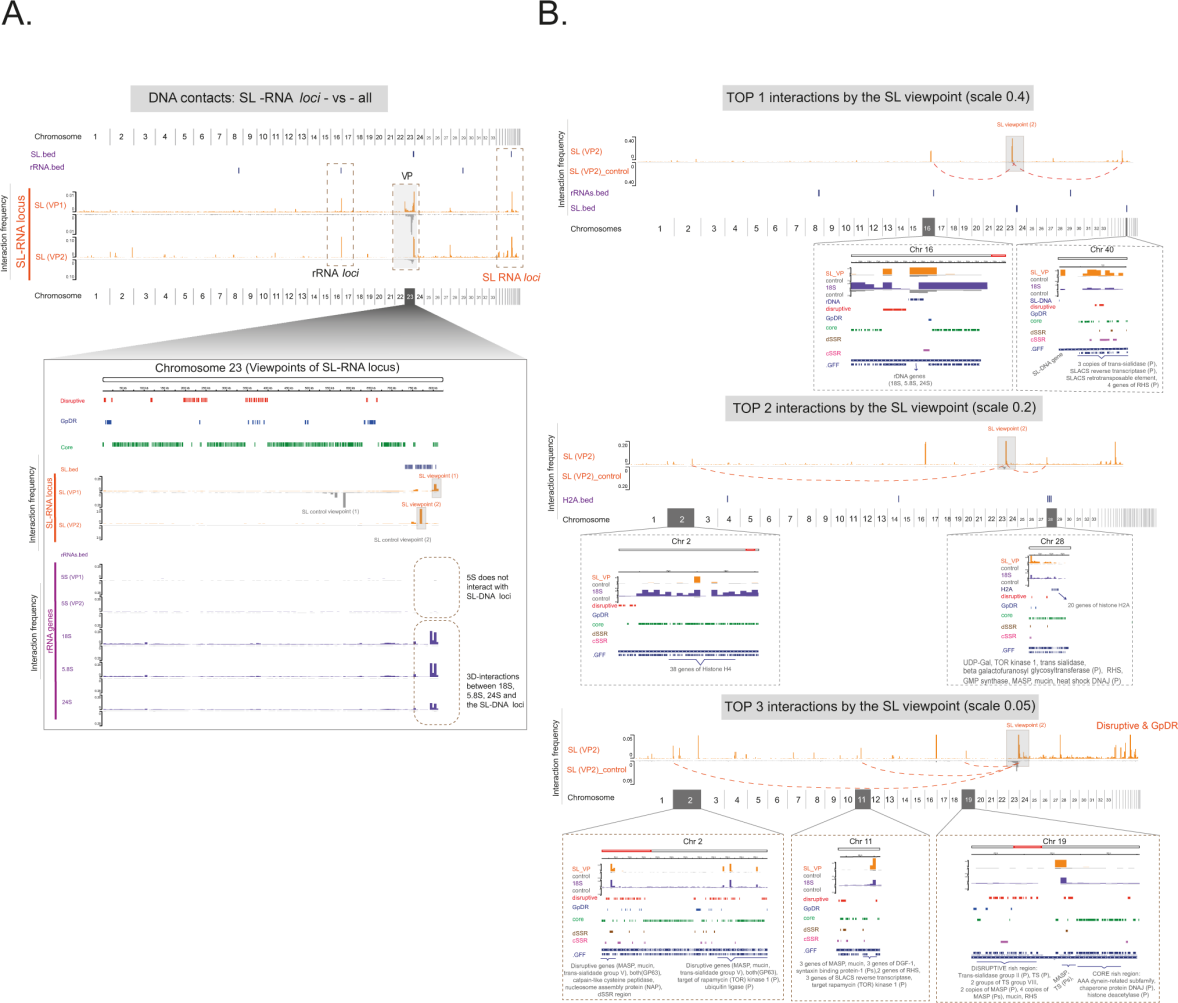
The 3D contacts involving the SL-RNA loci in *T. cruzi*. (A) Virtual 4C plot profile. The plot shows interaction frequencies of SL-RNA loci (VPs), orange plot, across the entire genome (the *x*-axis represents the genomic coordinates to all 43 chromosomes). Control viewpoints are included for comparison via flipped gray plots. A zoomed-in view of chromosome 23 depicts the viewpoints (VP1 and VP2) of origin for the SL-RNA loci, highlighting the interaction frequencies with the 18S rRNA, 5.8S rRNA, and 24S rRNA loci, excluding the 5S rRNA loci. (B) The top 1 to top 3 plots focused on the greatest number of DNA‒DNA interactions between SL-RNA loci and other genomic regions, indicating significant 3D nuclear architecture features.

The SL-RNA locus is also a highly transcribed gene essential for trans-splicing in trypanosomatids. It is transcribed by RNA polymerase II, and it contains a defined promoter region (2). Inspired by studies in *T. brucei* suggesting a role for SL-RNA loci in activating VSG genes through 3D proximity-based regulation (12), we assessed the 3D contact frequency for SL-RNA loci (VP on chromosome 23) with the rest of the genome by virtual 4C analysis (Fig. 6A). The SL-RNA loci frequently interact with the 18S, 5.8S, and 24S rRNAs (found at Chr 16) but not with the 5S rRNA loci (Chr 8 and 29), as shown in the VP profiles of the rRNA genes relative to the SL-RNA loci at Chr 23 (Fig. 6A, bottom).

We further identified seven main genomic regions exhibiting frequent interactions with the SL-RNA loci (Fig. 6B). The strongest interaction peaks included the rRNA locus (Chr 16), as mentioned above, and another SL-RNA locus (Chr 40) (Fig. 6B). The second level (top 2) of frequent interactions comprised the histone H4 array (38 gene copies on Chr 2), regions on chromosome 28 involving disruptive/*GpDR* genes (e.g., RHS, MASP, mucin), and conserved genes preceding the histone H2A array (20 gene copies) (Fig. 6B). The third most frequent interactions (top 3) include disruptive/*GpDR*-enriched genomic regions on chromosomes 2, 11, and 19 (Fig. 6B, third-ranking position), in addition to “Disruptive and *GpDR*” engagement located on the latest chromosomes. Additional 3D SL interactions with genes from the compartment disruptive or the *GpDR* group are shown in Fig. S8—peaks “d” to “h.”

**Fig 7:**
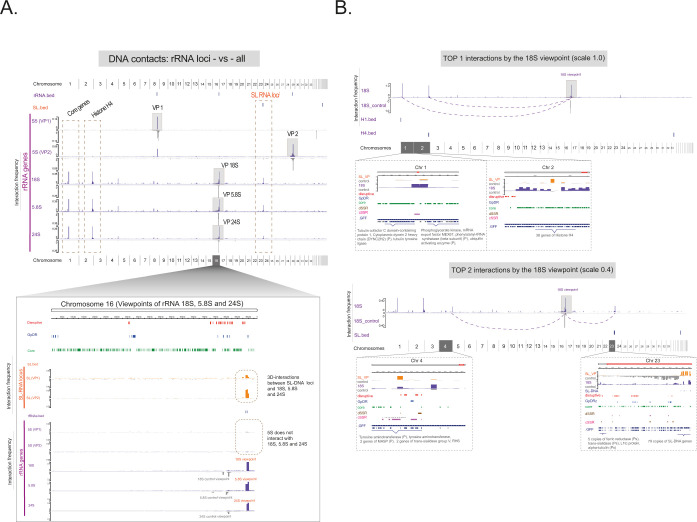
The 3D contacts involving the rRNA loci in *T. cruzi*. The plot profile highlights the virtual 4C results for the rRNA loci (VPs), purple plots, across the entire genome (the *x*-axis represents the genomic coordinates of all 43 chromosomes). Control viewpoints are included for comparison via flipped gray plots. A zoomed-in image of chromosome 16 shows the origin of the 18S rRNA, 5.8S rRNA, and 24S rRNA loci. SL-RNA locus interaction sites are highlighted, and no remarkable interactions are observed between the three rRNA genes (18S, 5.8S, and 24S) and the 5S rRNA locus. (B) The top (1 and 2) DNA‒DNA interactions involving the 18S rRNA locus. The figure includes annotations for genomic elements such as core genes and histone H4 regions.

Next, we investigated rRNA loci interactions motivated by their critical role in the translation machinery and nucleolar organization (50). We investigated the 3D interactions of 5S rRNA loci on chromosome 8 (68 gene copies) and chromosome 29 (1 gene copy) and for the 18S, 5.8S, and 24S rRNA gene arrays on chromosome 16. The spatial network between the SL-RNA array (Chr 23) and the 18S, 5.8S, and 24S loci, excluding the 5S RNA genes, was confirmed and evident at chromosome 16 (Fig. 7A).

Given that all rRNA genes on chromosome 16 exhibit similar whole-genome interaction profiles, we focused the analysis on the 18S viewpoint to inspect specific interactions (Fig. 7B). The histone H4 locus (Chr 2) showed a significant interaction frequency, sharing the top-ranking position with conserved genes on chromosome 1 involved in cell metabolism (e.g., phosphoglycerate kinase) and cytoskeleton assembly (e.g., tubulin- and dynein-related proteins). The second tier (top 2) of interactions includes the SL-RNA loci (Chr 23) and an array of disruptive/*GpDR* genes (Chr 4).

We noticed that a set of interactions is shared by both 18S and SL loci. We identified 34 peaks (labeled A to Z and a to h) in which 3 and 9 peaks are specific interactions of the SL-RNA loci and 18S rRNA loci, respectively (Fig. S8). For those peaks exclusive to 18S rRNA loci, the 3D contacts with the core genes are remarkable: cytoskeleton- and flagellum-related genes, heat shock genes (HSP70), and ribosomal subunits, among others (Fig. S8). In contrast, our 3D data indicate that disruptive and *GpDR* genes, which constitute approximately 17% and 7%, respectively, of all *T. cruzi* protein-coding genes (Fig. S2B, 1D view), interact more frequently with the SL-RNA loci (disruptive/*GpDR-*vs-SL-RNA) than with the 18S rRNA loci (disruptive/*GpDR-*vs-18S-rRNA) (Fig. 6 and Fig. S8, 3D view).

**Fig 8:**
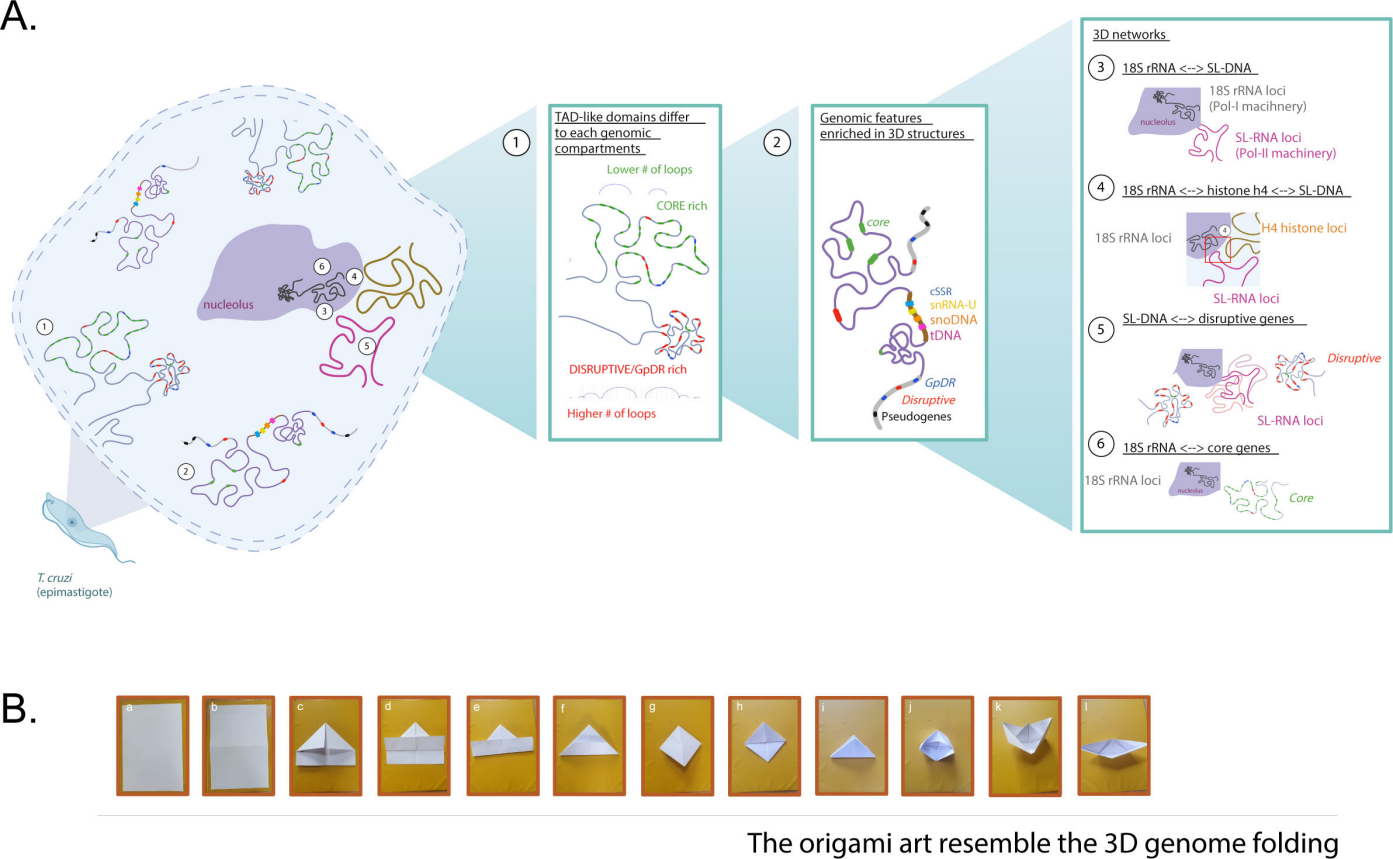
The spatial organization of chromatin within the nuclei of *T. cruzi* and its resemblance to origami art. (A) The non-random 3D nuclear architecture of *T. cruzi*. (1) Illustration of CF domains and loop formation, showing fewer loops in the core compartment and an increased number of loops in disruptive/GpDR genes. (2) Genomic features relevant to 3D structure formation. (3) Nucleolar organization: representation of the nucleolus, highlighting the localization of 18S rRNA loci (Pol I machinery) and SL-RNA loci (Pol II machinery). (4) DNA‒DNA interactions involving rRNA loci, the histone H4 array, and SL-RNA loci. (5) Prominent nuclear interaction networks in *T. cruzi* reveal frequent interactions between SL-RNA loci and disruptive/GpDR genes. (6) In contrast, core genes frequently interact with 18S rRNA loci. (B) Resemblance between origami art and chromatin folding. Steps “a” to “l” show the process of folding a flat piece of paper from its unidimensional view up to its 3D form.

Overall, our findings illustrate a strong 3D linkage between two main loci, the rRNAs and the SL-RNA array. Additionally, we revealed high frequency of contact of 18S rDNA loci (chromosome 16) and SL-RNA loci (chromosome 23) with the histone H4 array (chromosome 4) (Fig. 6 and 7). For the rRNA locus VP, other histone loci were also engaged in *trans*-acting contacts, such as histones H1 (Fig. S8, peak C), H2B and H2BV (Fig. S8, peak I), and H2A (Fig. S8, peak b). Together with the CF structure data, loop calling, and genomic feature enrichment in 3D structures, we demonstrated a non-random 3D organization of *T. cruzi* nuclei (summarized in Fig. 8).

## DISCUSSION

The *T. cruzi* genome, characterized by its high repetitive content (51.2%) that includes both non-coding and coding sequences (41), presents challenges for Hi-C read mapping. Repetitive regions often lead to low-quality mappings, which are often withdrawn during Hi-C processing. To overcome this issue, we used the mHi-C pipeline (45) to rescue high-quality Hi-C contacts in genomic regions enriched in repetitive DNAs. We unveiled that this strategy allowed comprehensive mapping and analysis of the 3D chromatin organization patterns of *T. cruzi* while also mitigating biases in the identification of CF domains, their boundaries, and the participation of genes from the MFs in these structures.

The MF of mucins, MASPs, and trans-sialidases play essential roles in cell invasion and immune response modulation during infection (51) and constitute approximately 29% of the *T. cruzi* repeatome (41). We observed that the MF genes, which exhibited lower transcriptional activity in noninfective forms (epimastigotes), formed shorter and more compact CF domains, while the core genes, with higher transcription rates and open chromatin levels (52), formed longer CF domains. Notably, the distinct genomic arrangement of these linear compartments is correlated with distinct gene activity levels and open chromatin profiles as previously reported (16, 35, 52). In addition, associations between the size of CF structures and genomic compartments have been previously reported (35), but these 3D regions were better elucidated because of the inclusion of multimapped reads reinforcing the higher compaction related to the disruptive compartment.

Our data indicated that chromosomes enriched in core genes had fewer chromatin loops than those enriched in disruptive/*GpDR* genes, which had at least twice as many loops, potentially associated with a gene regulatory mechanism. This organization mirrors *T. brucei* findings: lower DNA‒DNA contact frequency in core gene regions and greater frequency in subtelomeric regions (rich in VSG expression sites), indicating greater chromatin compaction in VSG-enriched areas (33). In *T. cruzi,* MF genes are dispersed throughout the genome but preferentially occupy unstructured 3D genomic regions. We hypothesize that certain segments of DNA may adopt a 3D conformation to safeguard loci that may not be essential during a specific life stage but could play crucial roles in other physiological scenarios. Therefore, further research is needed to better understand the relevance of this spatial organization to the parasite biology.

The 3D folding of chromatin is determined by various factors encoded in the linear genome. These include repetitive DNA elements, as well as DNA motifs for architectural proteins, promoters, and enhancers that form chromatin loops essential for gene regulation, and nonprotein-coding RNA loci (12, 39, 53, 54). Together, these elements help organize chromatin by connecting different genomic regions and shaping the intricate 3D structure of chromatin, impacting genomic architecture and function. In human and mouse genomes, tRNA loci mark TAD boundaries (46). In *Drosophila melanogaster*, the DNA motifs of architectural proteins and the open chromatin state define 3D structures (55). In *T. brucei*, the cohesin subunit sister chromatid cohesion 1 (SSC1) is enriched in tRNA genes and cSSRs (33). Here, we demonstrated that CF boundaries are also proportionally enriched (in length) in nonprotein-coding RNA loci, such as tRNAs, snoRNAs, and snRNAs, along with cSSRs—genomic regions not enriched in repetitive DNA content. In *T. cruzi*, tRNA and snRNA loci are linked to more open chromatin regions, especially in epimastigotes (replicative life form) (16). TAD-like boundaries are regions that restrict interactions of *cis*-regulatory sequences, contributing to the gene expression regulation of genes within TADs (46, 56). Although the PTU borders do not coincide perfectly with the CF boundaries, those that coincide may be functionally relevant. For those cases, it remains to be evaluated whether PTUs located in the same CF domain have similar transcription rates, since we detected variations in PTU transcription, particularly between PTUs from different genomic compartments (52).

TAD boundaries are enriched in DNA motifs for insulator proteins such as boundary element associated factor-32 and CTCF in flies and mammals, respectively (55, 57, 58). CTCF is a core architectural protein that establishes the 3D structure of many eukaryotic genomes (59). Since trypanosomes lack CTCF proteins, the identification of CF boundaries provided by our work has the potential to support further studies devoted to identifying regulatory proteins involved in 3D chromatin folding. This finding raises the possibility that trypanosomes harbor special alternative mechanisms to the well-known CTCF-based scheme encountered in most metazoans. Using the origami analogy, CTCF, cohesin, and similar proteins act like human fingers, precisely folding chromatin fibers into specific shapes.

Our data revealed that tRNA loci preferentially interact with one another in a 3D context, suggesting the formation of clusters that likely optimize transcription by RNA polymerase III, similar to what is observed in yeast (60). The improved *T. cruzi* genome annotation led to the 1D mapping of tRNA genes, highlighting clusters with two to seven genes in tandem, corroborating our hypothesis of RNA polymerase III transcription optimization. We identified a recurrent 3D co-localization of tDNAs with cSSRs, dSSRs, snoRNAs, and boundaries of CF domains, which together suggest a co-regulation of the RNA polymerase II and RNA polymerase III transcriptional machineries, as already claimed for yeast (61).

The SL-RNA loci predominantly interact in 3D with rRNA loci, with the histone H4 gene cluster, and with disruptive/*GpDR*-enriched genomic sites (mainly trans-sialidases, mucins, RHS). The SL-RNA loci interaction with surface-associated virulence factor genes suggests a potential regulatory role to enhance their expression, similar to what has been seen for VSG monoallelic expression in *T. brucei* (12). In this latter, a putative post-transcriptional enhancement was proposed as VSGs are transcribed by RNA Pol I, while both *T. cruzi* MFs and SL are transcribed by RNA Pol II. Therefore, we hypothesize that expression of surface-associated virulence factors in *T. cruzi* may take advantage of the proximity of RNA Pol II machineries enriched at SL loci (47) to boost both post-transcriptional and transcriptional mechanisms. Further investigations evaluating whether these 3D structures change in *T. cruzi* infective forms must be performed to better understand their impact on gene expression.

The nucleolus is an important subnuclear structure involved in rRNA synthesis and ribosome assembly and is critical to 3D genome organization (62). In mammals, nucleolus-associated chromatin interactions provide strong heterochromatin interactions (48). In *T. cruzi*, our study revealed that the 18S rRNA interactions include mainly core genes related to the cytoskeleton and flagellum, histone genes (H4, H1, H2B, H2BV, and H2A), ribosomal structural components, heat shock genes, and snoRNA genes (66 copies). These interactions suggest that *T. cruzi* nucleoli interactions involve transcribed/euchromatin regions (core genes) (52, 63) in contrast to other eukaryotes. Nevertheless, in addition to ribosomal biogenesis, the *T. cruzi* nucleolus extends its role by shaping the 3D chromatin structure, as observed in other eukaryotes (13). We hypothesize that the nucleolar disassembly in infective forms (22) may disrupt these interactions, potentially impacting their expression.

Some of the interactions highlighted here were previously detected, either directly or indirectly, using orthogonal techniques in trypanosomatids. For example, the main SL locus associated with the active VSG is also closer to the nucleolus in *T. brucei* (12), suggesting interactions between rRNA loci and SL-RNA loci. In *T. cruzi*, RNA polymerase II is concentrated near nucleoli and enriched at SL-RNA genes (47), also implying interactions between rRNA loci and SL-RNA loci. Our findings showing significant spatial contact between rRNA loci and SL-RNA loci and between SL-RNA loci and genes encoding surface-associated virulence factors from MFs confirm the previous observations. Notably, the 5S rRNA gene, transcribed by RNA Pol III, is distributed separately from the 18S rDNA/SL-DNA network, consistent with previous findings using fluorescent in situ hybridization(FISH) assays (64).

Although the computational approach used in our study to allocate multimapped reads (mHiC) has previously been shown to be robust, effective, and reproducible across a wide range of samples (including human, mouse, and *Plasmodium falciparum*) and read lengths (ranging from 36 bp to 151 bp) (45), we cannot exclude the possibility of misassignments in genomic regions shorter than the read length. Nevertheless, the precise chromosomal origin of some of these interactions is not critical, as our findings emphasize the biological significance of the interactions, such as those between the SL and rRNA loci, and GpDR/disruptive genes. We anticipate that integrating Hi-C with long-read sequencing approaches, such as pore-C (65), could more effectively address potential issues of misassigned regions in future studies.

The role of chromatin architecture in transcription control has been well documented across various organisms (66, 67). Previously, we found that the open chromatin status and nucleosome deposition were associated with mature (15, 16) and nascent transcript levels (52). Recently, the boundaries of CF domains were linked to a lack of mature RNA, leading to the assumption that trypanosome gene expression is determined by 3D organization (35). Here, we detected that identification of CF boundaries can be influenced by data processing with or without the inclusion of repeated genomic regions, which had not been taken into account previously (35). Our results suggest that the 3D nuclear architecture of *T. cruzi* might impact gene expression regulation through spatial interactions among different genomic loci, including the coordination of gene expression involving genomic loci transcribed by distinct RNA polymerases. The existence of 3D chromatin interactions may optimize cross-talk among RNA polymerase machineries, transcription factors, and epigenetic marks, which maintain or facilitate the transcription of target genomic regions.

## MATERIALS AND METHODS

### Data collection and read filtering

The Hi-C public data set from *T. cruzi,* Brazil A4 strain, available at the National Center for Biotechnology Information (NCBI) repository (SRA number SRX8355434) (34) was used in this work to characterize the 3D nuclear architecture profile. The quality of the paired-end raw Illumina FASTQ reads was assessed using FastQC v.0.12.1 (https://www.bioinformatics.babraham.ac.uk/projects/fastqc). The trimming of the adaptors from both forward and reverse sequenced reads, 5´ low-quality nucleotides removal, and removal of reads shorter than 70 bp in length were performed using Trimmomatic v.0.39 (68).

### mHi-C tool

The mHiC pipeline performs the Hi-C reads mapping while dealing with multimapped reads, followed by read ends pairing, valid fragment filtering, duplicate removal, genome binning, contact normalization, and matrix building. In total, six steps (S1 to S6) are performed by mHiC using six individual scripts—documented in reference 45. To suit the mHi-C scripts to the *T. cruzi* context (i.e., genome size and Hi-C paired-end read length), we based on the mHi-C scripts for *T. brucei* (https://github.com/bgbrink/mHiC). The two mapping steps (S1.1 and S1.4) included in the mHi-C pipeline were performed using the Burrows‒Wheeler Aligner (BWA) local alignment with the subcommand mem for mapping with the algorithm BWA-MEM, which is designed for longer sequences ranging from 70 bp to 1 Mbp. The parameter -SP5M was used following the 4D Nucleome guidelines for Hi-C mapping reads (https://data.4dnucleome.org/resources/data-analysis/hi_c-processing-pipeline). The first mapping step (S1.1) uses filtered R1 and R2 FASTQ files to map against the *T. cruzi* Brazil A4 reference genome (GenBank accession number GCA_015033625.1, version NCBI 2020). After the first mapping round (S1.1), multimapped reads that did not exceed 99 positions (filtered in the XA tag of the .sam file) were termed as high-quality reads. These reads were rescued, trimmed at the left 5´, and remapped (second mapping step—S1.4) onto the reference genome with the same BWA approach. In the S3 step, the reference genome was digested *in silico* considering DpnII restriction sites, and invalid Hi-C reads (e.g., self-circles, short-range interactions, self-ligations, dangling ends, and others of unknown origin, referred to as dump reads) were removed. Data normalization was performed using the Knight-Ruiz (KR) algorithm, and the genome was binned at a 5 Kbp resolution (S4 step). Then, the multimapped reads were converted into uniquely mapped reads filtering them with a posterior probability of 0.5 to assure that no more than one bin pair was used for each multiread pair, and the final mHi-C outputs were generated as binary .mHiC files (S6 step). The .mHiC matrices were converted into “.hic” files by an in-house script to enable their visualization with the JuiceBox interactive Hi-C contact matrix viewer (https://aidenlab.org/juicebox/).

### Hi-C explorer mapping, preprocessing, and matrix building

Mapping of the filtered R1 and R2 FASTQ files was performed as the S1.1 mHi-C step. Hi-C data preprocessing (44): the reference genome was digested *in silico* using the hicFindRestSite tool using “GATC” as the cut site to identify DpnII restriction sites, and a .bed file recording the restriction site positions along all the genome was obtained. Matrix building: the R1 and R2.sam files, output of the mapping step, and the .bed, output of the hicFindRestSite tool, were utilized for Hi-C contact matrix construction via the hicBuildMatrix tool, which generated a matrix containing the Hi-C contacts at a 0.5 Kbp bin resolution. Next, the hicMergeMatrixBins tool was used to merge neighboring genomic bins to obtain lower Hi-C map resolutions ranging from 2 to 20 Kbp. Normalization of all Hi-C pairwise matrices was performed using the KR method (69) to mitigate biases such as DNA compaction range, variations in GC content, and fluctuations in copy number by using the hicCorrectMatrix tool.

### TAD-like (CF domains) calling

CF domains and boundaries were identified using the hicFindTAD tool from HiCexplorer v.3.0. To determine the optimal settings for the *T. cruzi* genome (approximately 40 Mbp), we tested 2 Kbp and 5 Kbp matrices with deltas and thresholds ranging from 0.005 to 0.5. After evaluation, we selected a delta and threshold of 0.01 for both, as this configuration minimized spurious CF domain identification (data not shown).

### Chromatin loop calling

Chromatin loops were identified using the hicDetectLoops tool (70). Loop calling was conducted at the 2 Kbp resolution to compare loops in disruptive-rich chromosomes versus core-rich chromosomes.

### General feature format (GFF) improvement

#### ncRNA genes

Identification of small RNA genes was performed by BLASTn searches using the *T. cruzi* Dm28c genome-release54 (http://tritrypdb.org/) as the query, facilitated by a custom bash script called “findbestmatch” (available at https://github.com/trypchromics/tcruzi-origami). This script constructed BLAST indices for the *T. cruzi* Brazil A4 genome, executed BLASTn searches, and processed the .csv results to identify the best matches. Our analysis of the tRNA genes was compared with the tRNAScan web server (71). Additionally, a manual BLASTn search was conducted specifically for the selenocysteine tRNA gene using homologous sequences from *T. brucei* (Tb927.9.2380 and Tb27.9.2340). Box A and Box B sites were then identified for all tRNA genes using the strategy proposed by reference 72. The “findbestmatch” script was also used to identify snoRNAs, snRNAs, SL-RNAs, and rRNAs. Extragenomic regions: annotation of PTUs, transcription initiation sites (TSSs or dSSRs), termination sites (TTSs or cSSRs), and IRs was performed with the python-based “annotatePolycistron” script (https://github.com/alexranieri/annotatePolycistron).

### Assignment of target genes

#### Core/disruptive/GpDR

The GFF of *T. cruzi* Brazil A4 (NCBI version 2020) was screened to identify core and disruptive genomic compartments. Mucins, MASPs, and trans-sialidase genes were filtered out and designated as the disruptive .GFF file. The GP63, DGF-1, and RHS genes composed the GpDR .GFF file. The remaining filtered genes composed the core (housekeeping genes) .GFF. These files were further subdivided into “genes” and “pseudogenes.” All these groups were converted into .BED files for visualization with Integrative Genomics Viewer, and the percentage of target genes per chromosome was determined using the custom script “bedCoveragePerChrom” (available at https://github.com/trypchromics/tcruzi-origami).

#### Repetitive DNA

Based on the repeat list provided by reference 34, repeats shorter than 1,000 bp were excluded, resulting in 5,390 repetitive sequences grouped as DNAs (e.g., DNA/CMC-EnSpm, DNA/Zisupton), LINEs (e.g., LINE/CR1-Zenon, LINE/Jockey), low complexity A-rich regions, long terminal repeat (LTR) retrotransposons, rRNAs, simple repeats, satellites, and unknown types.

### 3D higher-order interactions between tRNA loci, rRNAs, and SL-RNA loci (virtual 4C)

The 4C approach, which uses the 3C principle combined with high-throughput sequencing, investigates DNA‒DNA interactions from a unique locus of interest (the VP) against the entire genome (73). Its virtual version, named virtual 4C, derives one-versus-all interactions from the genome-wide Hi-C matrix. In this study, the genomic coordinates of all tRNA genes formed 31 VPs after excluding overlaps (regions where a set of tRNA genes are arranged with less than 2 Kbp between each other). These regions were individually examined as VPs and input into the HiC Sunt Dracones package (https://github.com/foerstner-lab/HiCsuntdracones). The pipeline outputs a .wig file recording the set of Hi-C interactions constrained by each VP. The controls consisted of 31 genomic segments of similar size (bp) to the tRNA genes but located in different genomic regions. These genes were randomly sampled from the genome using the bedtools package, random function, with the options -l 73 (length), -n “variable” (number of control regions per chromosome, based on the number of real tRNA VPs per chromosome), and -seed 7135 (for shuffling). To determine the most likely 3D interactions, we calculated the mean Hi-C interactions for all tRNA genes, focusing on target genomic features (e.g., RNA loci, CF domains, boundaries, and extragenomic regions). An R script, based on the bedtools coverage function, was customized to calculate tRNA interactions with each target region. The virtual 4C signals were plotted and compared to those of random controls. The same method was applied to rRNA genes and SL-RNA loci for which we directly evaluated the .wig files, which were plotted in comparison to their corresponding control VPs.

## SUPPLEMENTAL MATERIAL

Supplemental material mbio.03861-24-s0001.pdf Supplemental figures and tables.

ASM does not own the copyrights to Supplemental Material that may be linked to, or accessed through, an article. The authors have granted ASM a non-exclusive, world-wide license to publish the Supplemental Material files. Please contact the corresponding author directly for reuse.
