# Supplementary material for: Hidden origami in Trypanosoma cruzi nuclei highlights its non-random 3D genomic organization
Source: mBio. 2025 Apr 17;16(5):e03861-24. doi: 10.1128/mbio.03861-24 (PMC12077095; doi:10.1128/mbio.03861-24)
Supplement: Supplemental material — Supplemental figures and tables. [file mbio.03861-24-s0001.pdf]

Supplemental material for

"Hidden origami in *Trypanosoma cruzi* nuclei highlights its non-random 3D genomic organization"

Natália Karla Bellini[a,b], Pedro Leonardo Carvalho de Lima[a,b], David da Silva Pires[a,b], Julia Pinheiro Chagas da Cunha[a,b,#]

a. Cell Cycle Laboratory, Butantan Institute, São Paulo, Brazil
b. Center of Toxins, Immune Response and Cell Signaling (CeTICS), Butantan Institute, São Paulo, Brazil

Running title: The non-random 3D nuclear organization of *T.cruzi*

# Address correspondence to Julia PC da Cunha, julia.cunha@butantan.gov.br

Table S1. Improvement of *T. cruzi* Brazil A4 GFF file by annotating RNA loci across the genome. Results for the identification of small RNA genes by using the "findBestMatch" script showing the RNA type, number of genes, and genomic locations across chromosomes and contigs.

Table S2. Identification of tRNA genes in the *T. cruzi* Brazil A4 genome. Comparison between "findBestMatch" algorithm and tRNA_Scan outputs presenting the genomic positions, corresponding amino acids, and distribution of tRNA genes across chromosomes and contigs. Additionally, a manual BLASTn search targeting the selenocysteine (Sec) tRNA gene is included.

Table S3. Increase in Hi-C contacts for dry-bench analysis including repetitive DNA. Comparison of QC report statistics for mapping and building the Hi-C matrices using the Hi-CExplorer (A) and the mHi-C (B) pipelines emphasizing the impact (12.5 million reads gain) of accounting for repetitive DNA in the analysis of Hi-C data.

Fig S1. Distribution of tRNA genes in *T. cruzi* Brazil A4 genome. A. Consensus sequences for the tRNA gene promoter elements (A and B boxes) used for sense/antisense identification as per Marck et al., 2006 (1). B. SnapGene snapshot depicting the identification of A and B boxes in two target tRNA genes identified in *T. cruzi* Brazil A4 using the "findBestMatch" script. C. Illustration of the genomic locations of tRNA genes across the *T. cruzi* Brazil A4 genome. VP1 to VP31 represent the viewpoint positions used for virtual4C analysis to investigate DNA–DNA interactions mediated by tRNAs within *T. cruzi* nuclei.

Fig S2. Composition of genes and pseudogenes from each genomic compartment, as well as, repetitive DNA, in the *T. cruzi* Brazil A4 genome. A. Percentages of genes and pseudogenes belonging to the *core*, *disruptive* and GpDR compartments across the 43 chromosomes. B. Overall percentages of genes and pseudogenes across the genomic compartments highlighting that GpDR and *disruptive* are abundant in pseudogenes. C. Abundance (in percentage, based in length) of repetitive DNA and pseudogenes across the 43 chromosomes. D. The top bar graph represents the composition (in percentage, y-axis) of the *core* (green), *disruptive* (red), and *GpDR* (blue) genes per chromosome (x-axis). The bottom bar graph displays the chromosome size distribution, in length (Mbp). Pink dashed rectangles highlight pairs of chromosomes comparable in length but with contrasting compositions in *core* versus *disruptive*/*GpDR* content evaluated in loop calling analysis.

Fig S3: Evaluation of repetitive DNA content in *T.cruzi* genome. A - Bar plots show the percentage of enrichment of genomic features in the repetitive DNA. Acronyms meaning: Multigenic family of genes (MFs), cSSR and dSSR (convergent and divergent strand switch

regions), intergenic regions (ITR), sire (short interspersed repetitive element), L1tc (long autonomous retroposons of the ingi clade), LTR (long terminal repeat), SL (spliced-leader genes). B - Global alignment of nonprotein coding genes performed with SnapGene tool using the Clustal W algorithm. Yellow nucleotides represent >90% conservation and highlight that the genes within the groups 5S and SL DNAs share higher conservation.

Fig S4. Hi-C matrix resolution effect in TAD (CF domains) calling for *T. cruzi* Brazil A4 genome. A. Identification of CF domains (black triangles - x-axis) in KR-normalized Hi-C heatmaps for chromosome 6 according to matrix resolution ranging from 2 to 20 Kbp (y-axis). B. Pie charts representing the overall (43 chromosomes) genomic coverage percentages of the CF domains, boundaries, and unstructured regions according to the matrix resolution. C. Variation in CF domains length across different Hi-C matrix resolutions. The white numbers inside the boxplots represent the CF median lengths. CF domains illustrations are shown above the boxplots. D. The Hi-C matrix of chromosome 3 shows overlapping and nested CF domains identified at 20 Kbp, 10 Kbp, 5 Kbp and 2 Kbp resolution. This figure illustrates how increasing the resolution of the Hi-C matrix leads to a decrease in the median length of TAD-like domains.

Fig S5. Variation in CF domain lengths (bp) across genomic compartment. Boxplot showing the length (bp) distributions of the *core* (green), *disruptive* (red), and *GpDR* (blue)-enriched CF domains at a 2 Kbp matrix resolution. The significance levels (* and ****) indicate the results of the Wilcoxon–Mann–Whitney test with p values < 0.5 and 0.001, respectively.

Fig S6. Analysis of CF domains and boundaries comparing the exclusion versus inclusion of multimapping reads in the Hi-C data analysis. A. Number of CF domains and boundaries across the 43 chromosomes of *T. cruzi* Brazil A4. B. Comparison of CF domains length (Kbp) for each dry-bench approach. C. Bar plot showing the bias of boundary detection in repetitive DNA-rich regions when multimapped reads are not included (HiCExplorer).

Fig S7. Unstructured regions are enriched in *disruptive*/*GpDR* genes, as well as repetitive DNA. Integrative Genomics Viewer (IGV) snapshots for chromosomes 5, 7 and 19 highlighting the coincidence between unstructured regions of the genome (pink dashed rectangles), repetitive DNA (orange track), *disruptive* (red track) and *GpDR* (dark blue track) genes. This analysis includes CF domains and their boundaries (light blue tracks) identification in Hi-C matrices as accounting for DNA–DNA contacts in repetitive DNA regions.

Fig S8. 3D interactions involving 18S rRNA loci and SL-RNA loci within the *T. cruzi* nucleus. This panel provides a finer resolution of interaction hotspots involving both SL-RNA loci and 18S rRNA loci and their individual interactions, accompanied by the genomic context surrounding each interaction. The upper plots show the interaction profiles for the SL (orange) and 18S (purple) loci viewpoints. The letters above each peak indicate shared (brown) or individual interactions for each viewpoint (orange to SL and purple to 18S). The zoomed panel shows IGV snapshots highlighting the genomic context of each peak, from A to Z and a to h, with the individual interactions indicated between parentheses. It illustrates the complex 3D interaction landscape involving key RNA genes in the *T. cruzi* nucleus, providing detailed insights into the spatial organization and interaction dynamics of the SL-RNA and 18S rRNA genes.

References

1. Marck C, Kachouri-Lafond R, Lafontaine I, Westhof E, Dujon B, Grosjean H. 2006. The RNA polymerase III-dependent family of genes in hemiascomycetes: Comparative RNomics, decoding strategies, transcription and evolutionary implications. Nucleic Acids Res 34:1816–1835.

RNAs loci annotation			
<u>RNA type</u>	<u>Subtypes</u>	<u># of genes</u>	<u>Genomic Location</u>
**tRNA**		71	Chrms (1, 10, 14, 16, 17, 19, 2, 22, 34, 4, 5, 7, 8), Contig 330
**snoRNA**	snoRNA-HACA	332	Chrms (1, 11, 12, 13, 15, 19, 26, 27, 3, 32, 34, 4, 41, 6, 8, 9), Contigs(107,130,155,221,258,263,303,312,32,344,347,378,394)
	snoRNA-CD	867	Chrms (1, 10, 11, 12, 13, 14, 15, 17, 2, 22, 24, 25, 26, 27, 3, 31, 32, 33, 34, 35, 38, 39, 4, 41, 42, 43, 5, 6, 7, 8, 9), Contigs (1, 107, 118, 130, 135, 139, 152, 155, 157, 159, 167, 199, 203, 220, 221, 229, 258, 26, 263, 268, 303, 307, 312, 318, 32, 322, 327, 333, 340, 344, 347, 352, 37, 378, 380, 390, 394, 4, 54, 57, 95)
**ncRNA**	SL	96	Chrms (23 and 40), Contigs (112,113, 62)
	snRNA_U1	1	Chrm 17
	snRNA_U2	3	Chrms (20, 4, 5)
	snRNA_U3	1	Chrm 17
	snRNA_U4	1	Chrm 34
	snRNA_U5	1	Chrm 4
	snRNA_U6	2	Chrm 10
**rRNA**	18S	13	Chrm 16, Contigs (126,13,136,160,2,279,297,339,59)
	5S	92	Chrms (8 and 29), Contig 400
	5.8S	13	Chrms 16, Contigs (13,136,160,2,279,297,323,59)
	24S_s1	16	Chrm 16, Contigs (126,13,136,160,2,279,296,297,310,323,339,59)
	24S_s2	14	Chrm 16, Contigs (13,126, 136,2,279,296,297,310,323,339,351,59)
	24S_s4	10	Chrm 16, Contigs (126,13,136,297,310,323,339,351,59)
	24S_s6	15	Chrm (16), Contigs (13,126, 136,2,279,296,297,310,323,339,351,59)
	24S-alpha	14	Chrm 16, Contigs (13,136,160,2,279,296,297,323,339,59)
	24S-beta	14	Chrms (16), Contigs (13,126, 136,160,2,296,297,310,323,339,59)

Supplementary Table S1

tRNA genes annotation							
tRNA_scan result				0-findBestMatch result			
	Genomic Position				Genomic Position		
ID	Start	End	Aminoacid	ID	Start	End	Aminoacid
TcBrA4_Chr1	254760	254831	Pro	TcBrA4_Chr1	254760	254831	Pro
TcBrA4_Chr1	1630559	1630630	Cys	TcBrA4_Chr1	1630559	1630630	Cys
TcBrA4_Chr2	370449	370522	Ile	TcBrA4_Chr2	1093858	1093930	Ala
TcBrA4_Chr2	1093555	1093627	Phe	TcBrA4_Chr2	1137246	1137318	Ala
TcBrA4_Chr2	634861	634933	Val	TcBrA4_Chr2	1093732	1093803	Arg
TcBrA4_Chr2	1137373	1137444	Arg	TcBrA4_Chr2	1137373	1137444	Arg
TcBrA4_Chr2	1137247	1137318	Ala	TcBrA4_Chr2	635158	635230	Phe
TcBrA4_Chr2	635023	635094	Glu	TcBrA4_Chr2	1093555	1093627	Phe
TcBrA4_Chr2	1093732	1093803	Arg	TcBrA4_Chr2	1137550	1137622	Phe
TcBrA4_Chr2	1093858	1093929	Ala	TcBrA4_Chr2	635023	635094	Glu
TcBrA4_Chr2	635158	635230	Phe	TcBrA4_Chr2	634861	634933	Val
TcBrA4_Chr2	1137550	1137622	Phe	TcBrA4_Chr2	155591	155664	Ile
TcBrA4_Chr2	155591	155664	Ile	TcBrA4_Chr2	370449	370522	Ile
TcBrA4_Chr4	837258	837329	Ala	TcBrA4_Chr4	837143	837215	Asn
TcBrA4_Chr4	209398	209469	Pro	TcBrA4_Chr4	837258	837329	Ala
TcBrA4_Chr4	209546	209617	Met	TcBrA4_Chr4	837417	837489	Arg
TcBrA4_Chr4	837547	837618	Lys	TcBrA4_Chr4	837547	837618	Lys
TcBrA4_Chr4	837143	837215	Asn	TcBrA4_Chr4	837684	837756	Arg
TcBrA4_Chr4	837417	837489	Arg	TcBrA4_Chr4	209398	209469	Pro
TcBrA4_Chr4	837684	837756	Arg	TcBrA4_Chr4	209546	209617	Met
TcBrA4_Chr4	209677	209758	Leu	TcBrA4_Chr4	209677	209758	Leu
TcBrA4_Chr5	545457	545528	Arg	TcBrA4_Chr5	545457	545528	Arg
TcBrA4_Chr7	554922	555003	Leu	TcBrA4_Chr7	538708	538788	Ser
TcBrA4_Chr7	555060	555140	Ser	TcBrA4_Chr7	555060	555140	Ser
TcBrA4_Chr7	538708	538788	Ser	TcBrA4_Chr7	554922	555003	Leu
TcBrA4_Chr8	693545	693626	Leu	TcBrA4_Chr8	693545	693626	Leu
TcBrA4_Chr8	693708	693778	Gly	TcBrA4_Chr8	693708	693778	Gly
TcBrA4_Chr10	176568	176648	Ser	TcBrA4_Chr10	792803	792875	Asn
TcBrA4_Chr10	175887	175960	Val	TcBrA4_Chr10	1087763	1087835	Asn
TcBrA4_Chr10	1087763	1087835	Asn	TcBrA4_Chr10	176731	176810	Leu
TcBrA4_Chr10	792803	792875	Asn	TcBrA4_Chr10	176568	176648	Ser
TcBrA4_Chr10	176170	176242	Met	TcBrA4_Chr10	176170	176242	Met
TcBrA4_Chr10	1087910	1087981	Thr	TcBrA4_Chr10	176467	176539	Met
TcBrA4_Chr10	792950	793021	Thr	TcBrA4_Chr10	176314	176385	Glu
TcBrA4_Chr10	176314	176385	Glu	TcBrA4_Chr10	176056	176141	Tyr
TcBrA4_Chr10	793283	793354	Pro	TcBrA4_Chr10	175887	175960	Val
TcBrA4_Chr10	1088242	1088313	Pro	TcBrA4_Chr10	792950	793021	Thr
TcBrA4_Chr10	176467	176539	Met	TcBrA4_Chr10	1087910	1087981	Thr
TcBrA4_Chr10	176731	176810	Leu	TcBrA4_Chr10	793283	793354	Pro
TcBrA4_Chr10	176056	176141	Tyr	TcBrA4_Chr10	1088242	1088313	Pro
TcBrA4_Chr14	736285	736356	Asp	TcBrA4_Chr14	736285	736356	Asp
TcBrA4_Chr14	260465	260535	His	TcBrA4_Chr14	736462	736542	Ser
TcBrA4_Chr14	262720	262790	His	TcBrA4_Chr14	736613	736685	Ala

Supplementary Table S2

TcBrA4_Chr14	736613	736685	Ala	TcBrA4_Chr14	260465	260535	His
TcBrA4_Chr14	736462	736542	Ser	TcBrA4_Chr14	262720	262790	His
TcBrA4_Chr16	798326	798398	Undet	TcBrA4_Chr16	782085	782157	Val
TcBrA4_Chr16	782085	782157	Undet	TcBrA4_Chr16	798326	798398	Val
TcBrA4_Chr16	828368	828439	Glu	TcBrA4_Chr16	828368	828439	Glu
TcBrA4_Chr17	482832	482904	Val	TcBrA4_Chr17	483128	483201	Ile
TcBrA4_Chr17	482689	482761	Lys	TcBrA4_Chr17	482689	482761	Lys
TcBrA4_Chr17	481402	481474	Lys	TcBrA4_Chr17	481402	481474	Lys
TcBrA4_Chr17	482971	483042	Gln	TcBrA4_Chr17	481288	481359	Arg
TcBrA4_Chr17	482365	482436	Gly	TcBrA4_Chr17	482971	483042	Gln
TcBrA4_Chr17	481946	482017	Arg	TcBrA4_Chr17	482832	482904	Pro
TcBrA4_Chr17	361038	361109	Trp	TcBrA4_Chr17	482365	482436	Gly
TcBrA4_Chr17	481288	481359	Arg	TcBrA4_Chr17	482242	482323	Leu
TcBrA4_Chr17	482106	482177	Thr	TcBrA4_Chr17	482106	482177	Gly
TcBrA4_Chr17	483128	483201	Ile	TcBrA4_Chr17	481946	482017	Arg
TcBrA4_Chr17	482242	482323	Leu	TcBrA4_Chr17	361038	361109	Trp
TcBrA4_Chr19	155546	155618	Undet	TcBrA4_Chr19	24114	24186	Val
TcBrA4_Chr19	24114	24186	Undet	TcBrA4_Chr19	155546	155618	Val
TcBrA4_Chr22	477521	477602	Leu	TcBrA4_Chr22	477822	477895	Ile
TcBrA4_Chr22	613988	614059	Gln	TcBrA4_Chr22	477521	477602	Leu
TcBrA4_Chr22	477662	477733	Gln	TcBrA4_Chr22	477662	477733	Gln
TcBrA4_Chr22	477822	477895	Ile	TcBrA4_Chr22	613988	614059	Gln
TcBrA4_Chr34	141387	141457	Gly	TcBrA4_Chr34	108934	109004	Gly
TcBrA4_Chr34	108934	109004	Gly	TcBrA4_Chr34	141387	141457	Gly
TcBrA4_Chr34	109086	109157	Thr	TcBrA4_Chr34	109086	109157	Thr
TcBrA4_Contg330	77803	77875	Phe	TcBrA4_Contig33(	78108	78180	Ala
TcBrA4_Contg330	77981	78052	Arg	TcBrA4_Contig33(	77981	78052	Arg
TcBrA4_Contg330	78108	78179	Ala	TcBrA4_Contig33(	77803	77875	Phe

Mannual BLASTn search (tDNA_Sec)			
	Genomic Position		
ID	Start	End	Aminoacid
TcBrA4_Chr27	503119	503206	Sec
TcBrA4_Chr27	506610	506697	Sec
TcBrA4_Chr27	510085	510172	Sec
TcBrA4_Chr27	513577	513665	Sec
TcBrA4_Chr27	517083	517170	Sec
TcBrA4_Chr27	520572	520659	Sec

Supplementary Table S2

A. **Multimapping reads discarded (HiCExplorer)**

	Number of Reads	Percentage
**Total**	361942919	
**Unmapped**	58401665	16%
**Singleton**	100878479	28%
**Mapped**	202662775	56%
**Uniquely**	134483874	66%
**Multimapping**	68178901	34%

134483874: total reads used to build Hi-C matrix

B. **Multimapping reads included (mHiC)**

	**Number of Reads**	**Percentage**
**Total**	361942919	
**Unmapped**	88434911	24%
**Singleton**	122329846	34%
**Mapped**	151178162	42%
**Uniquely**	77131922	51 %
**Multimapping**	74046240	49%
**High quality**	69882973	94%
**Low quality**	4163267	6%

147014895: total reads used to build Hi-C matrix (gain of about 12.5 M reads)

Supplementary Table S3

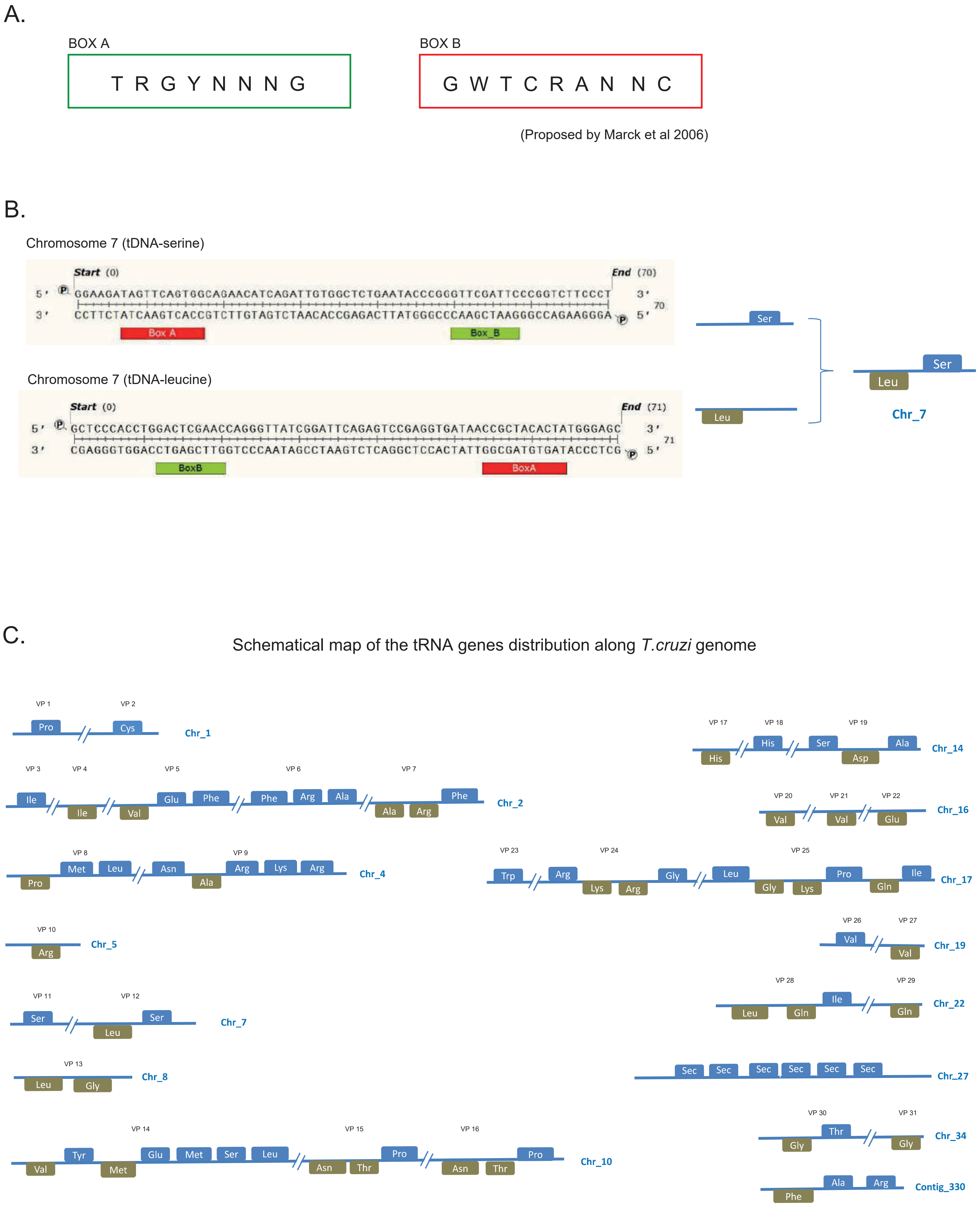

Supplementary Figure S1

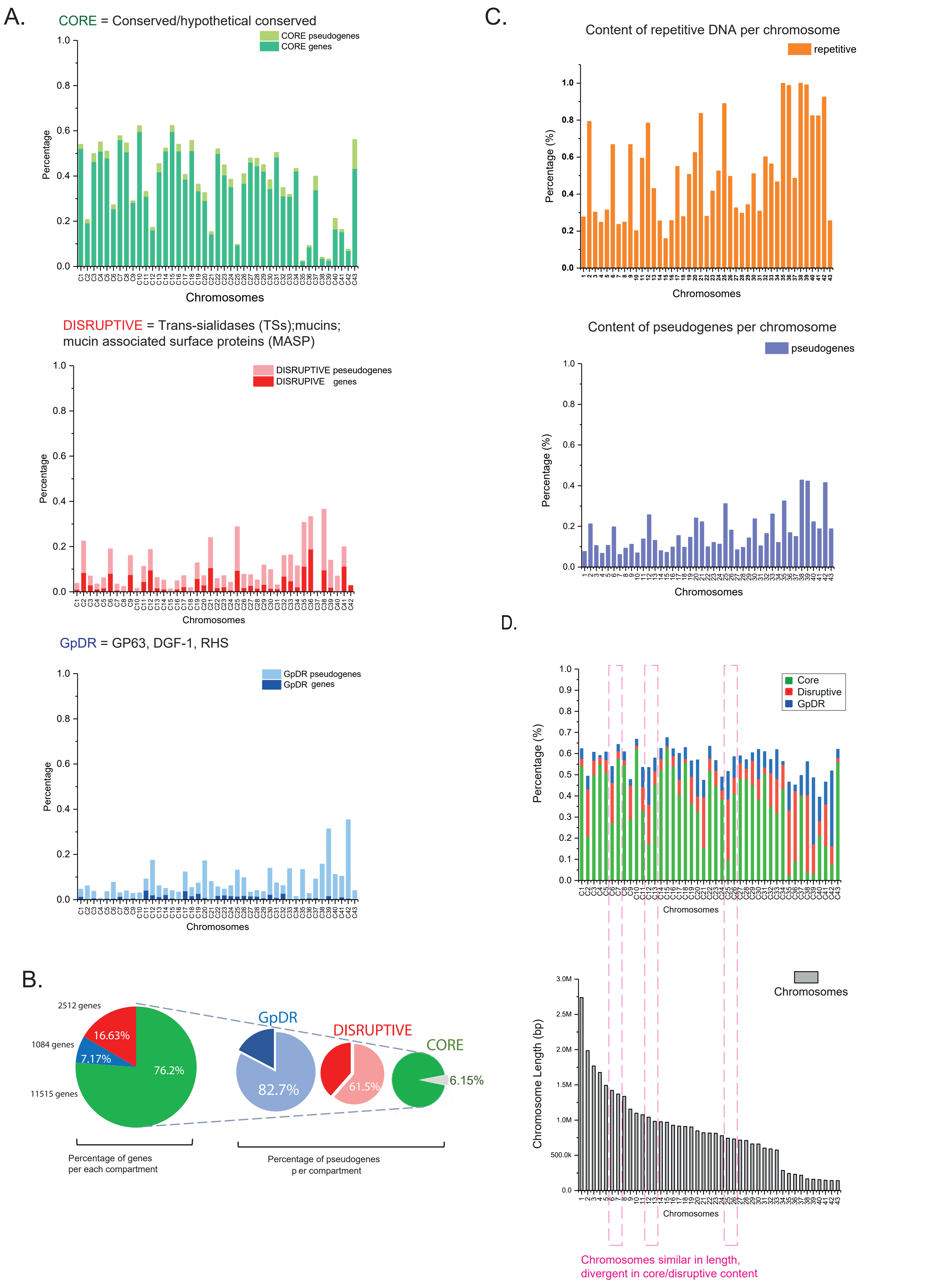

Supplementary Figure S2

A.

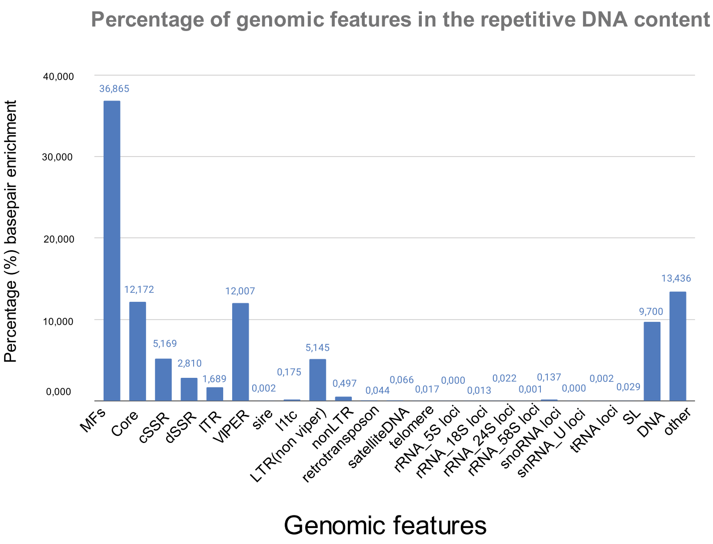

B.

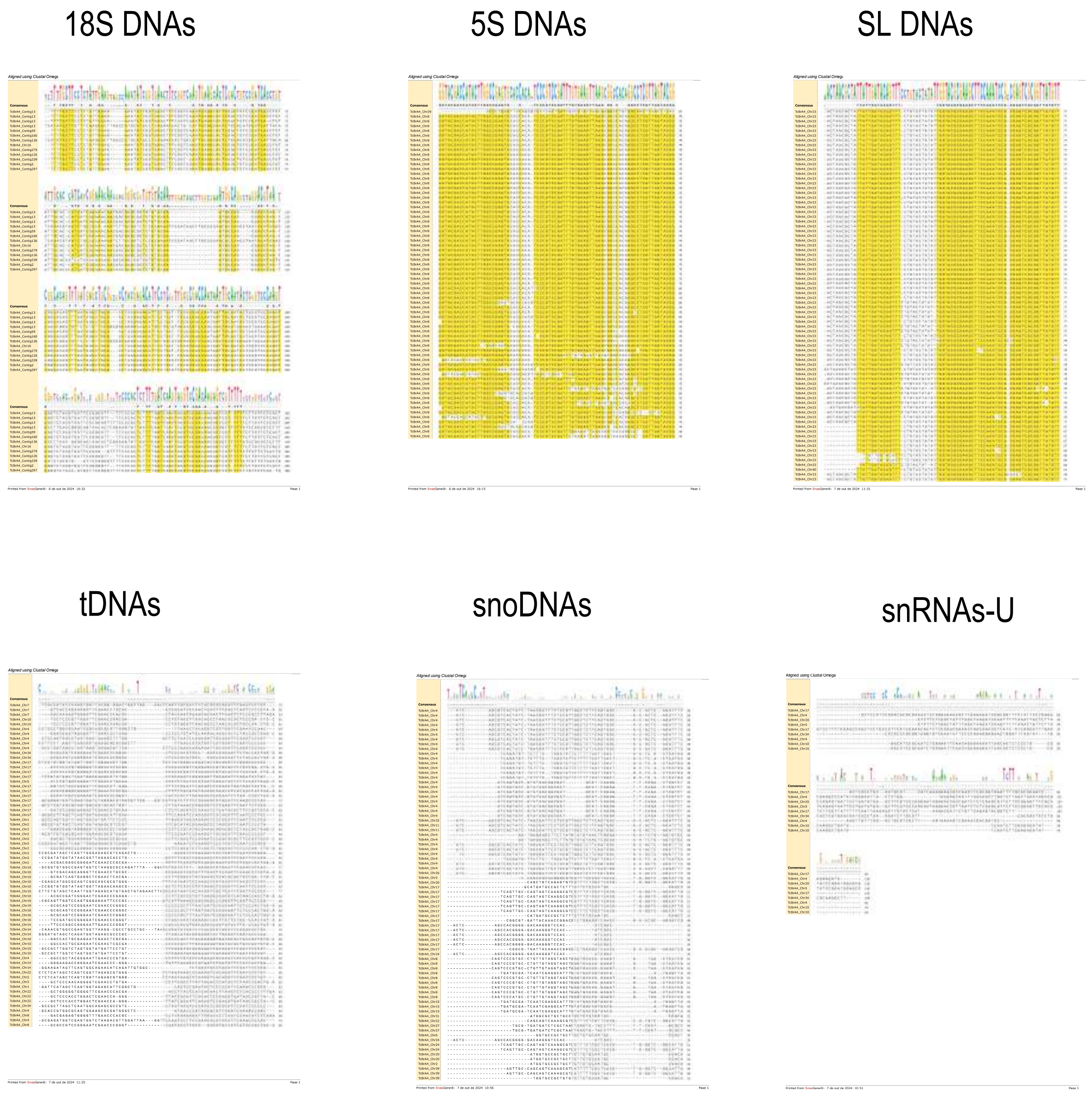

Supplementary Figure S3

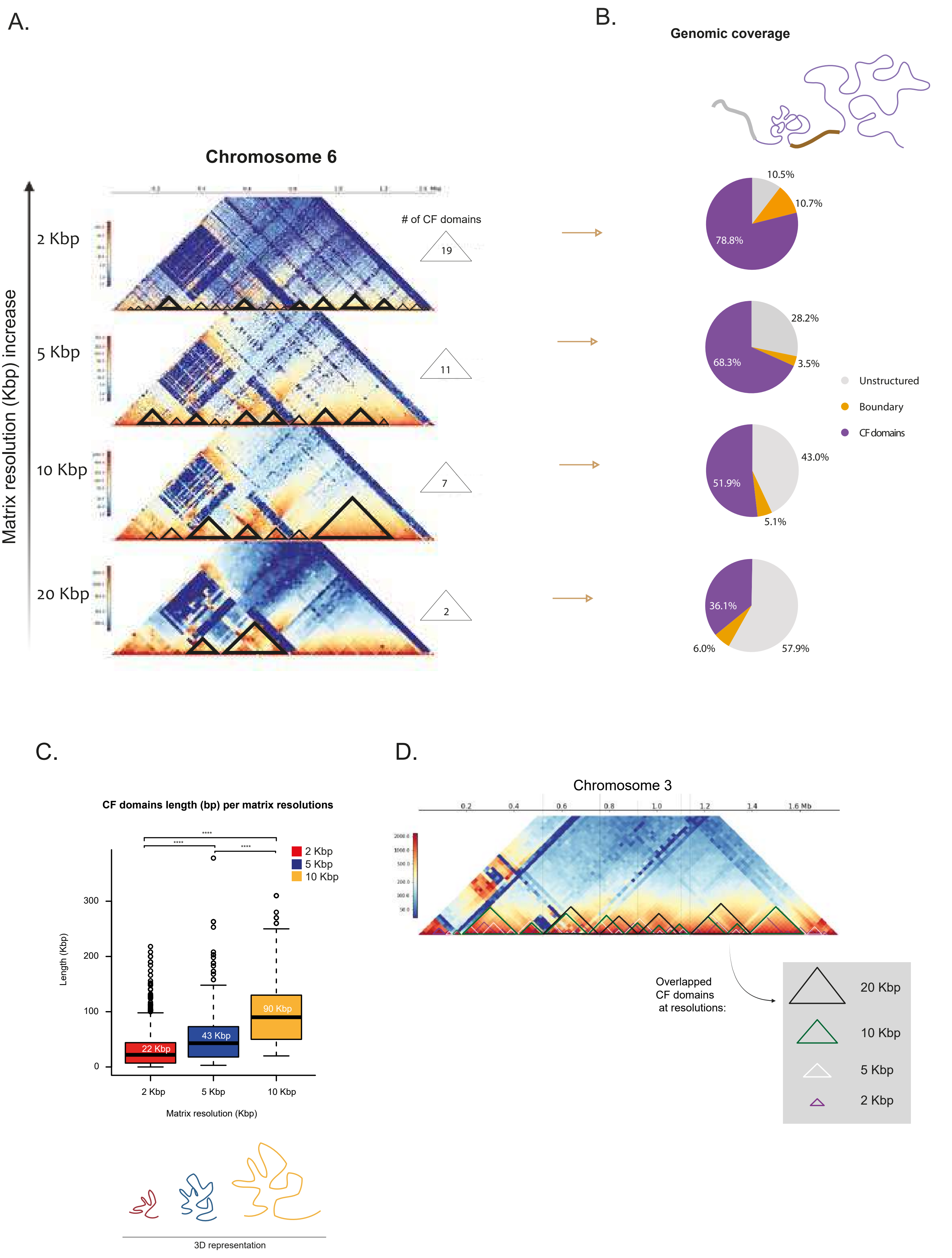

Supplementary Figure S4

Distribution of CF domain length (bp) across genomic compartments

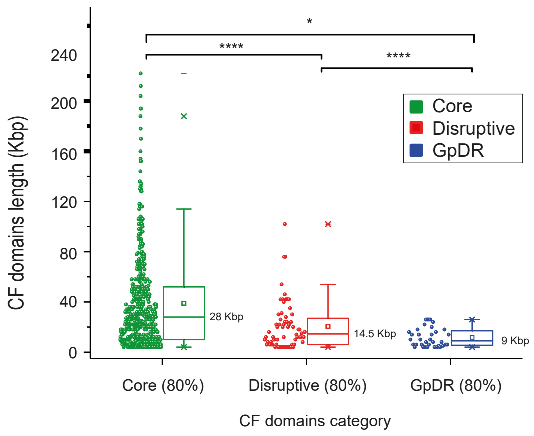

Supplementary Figure S5

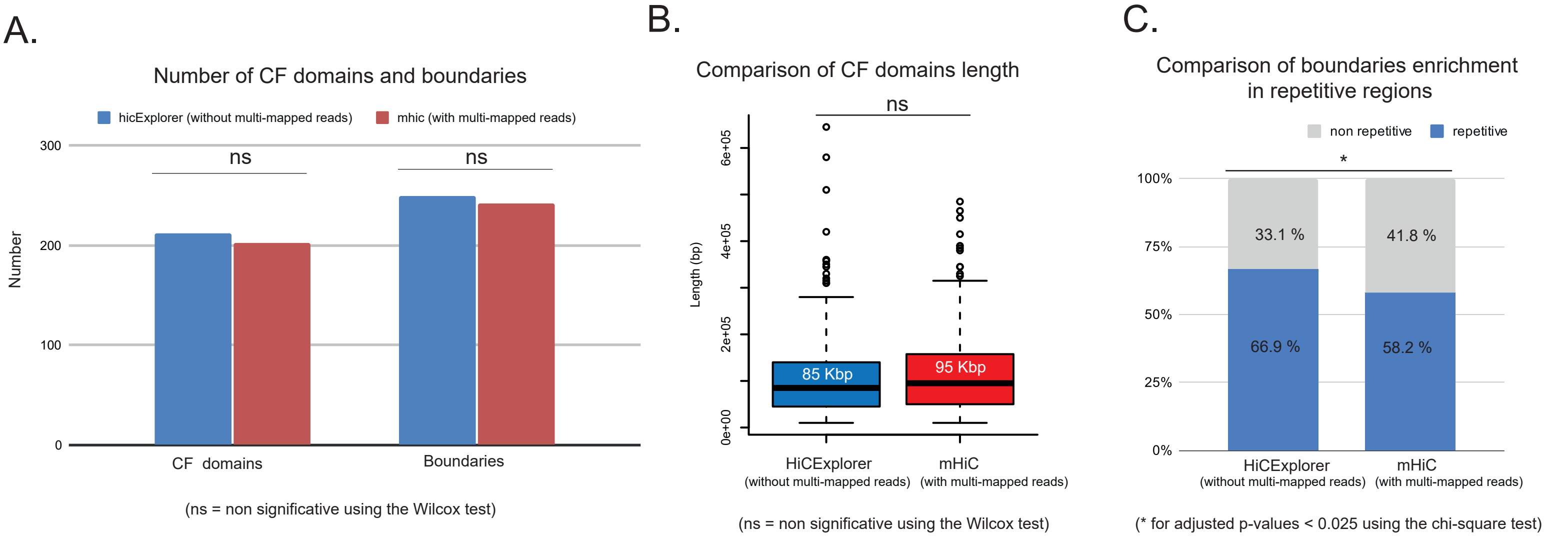

Supplementary Figure S6

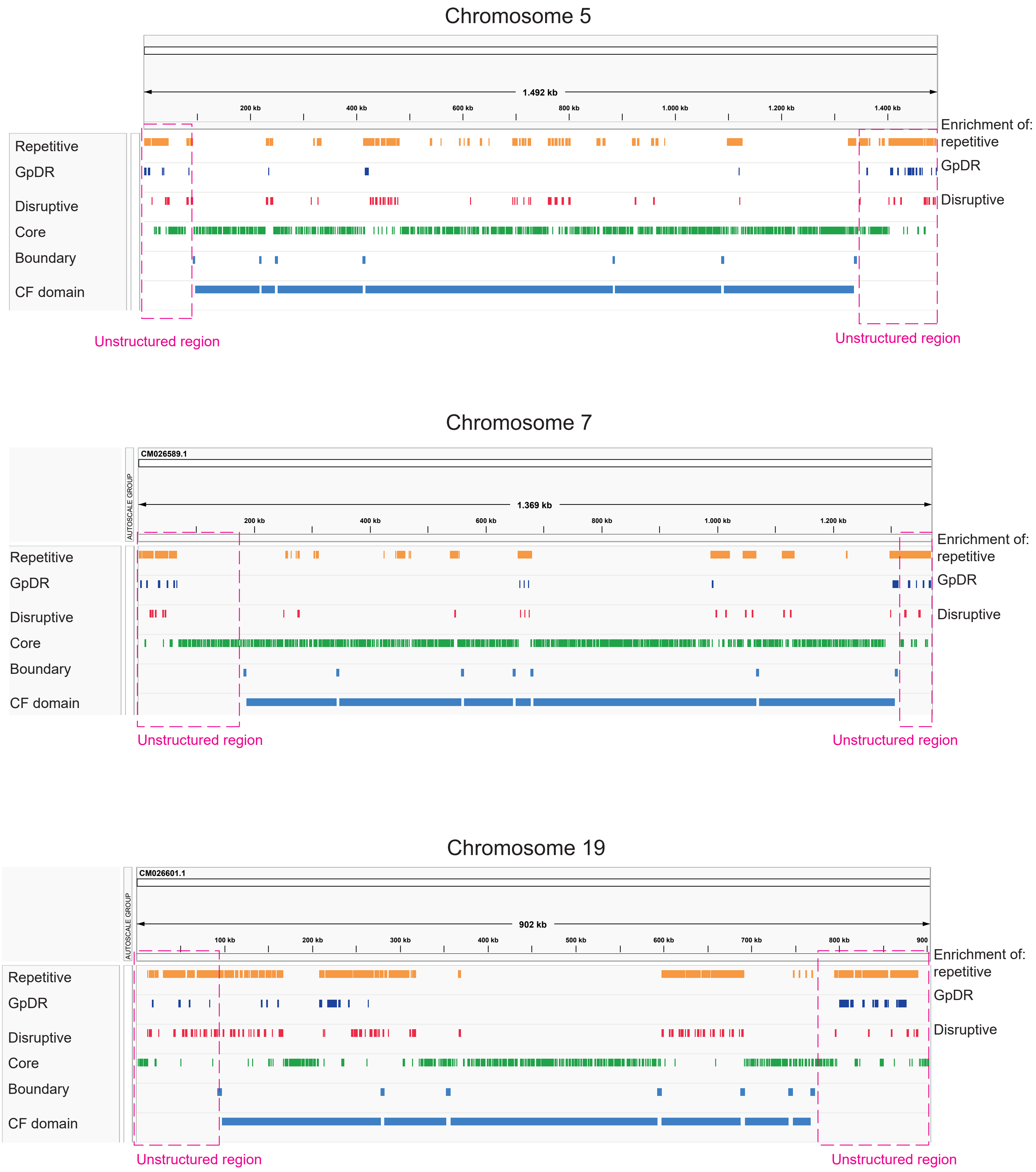

Supplementary Figure S7

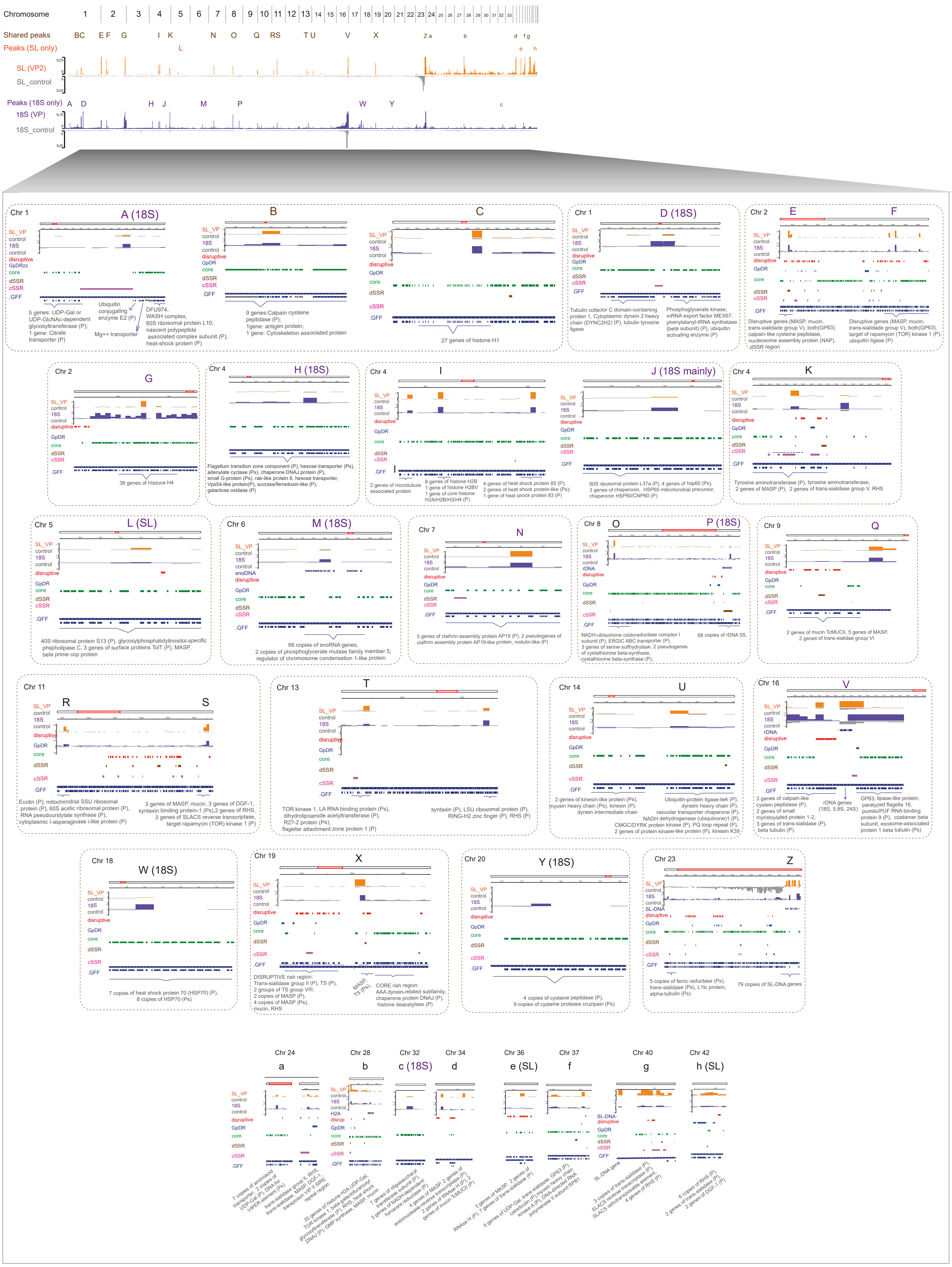

Supplementary Figure S8
